# Micropatterning and Nanodropletting of Titanium by Shifted Surface Laser Texturing Significantly Enhances In Vitro Osteogenesis of Healthy and Osteoporotic Mesenchymal Stromal Cells

**DOI:** 10.3390/jfb16110401

**Published:** 2025-10-27

**Authors:** Theresia Stich, Francisca Alagboso, Girish Pattappa, Jin Chu, Denys Moskal, Michal Povolný, Maximilian Saller, Veronika Schönitzer, Konstantin J. Scholz, Fabian Cieplik, Volker Alt, Maximilian Rudert, Tomáš Kovářík, Tomáš Křenek, Denitsa Docheva

**Affiliations:** 1 Experimental Trauma Surgery, Department of Trauma Surgery, University Regensburg Medical Centre, 93055 Regensburg, Germany; 2 Department of Musculoskeletal Tissue Regeneration and Department of Orthopedic Surgery, Orthopedic Hospital König-Ludwig-Haus, Julius-Maximilians-University Würzburg, 97074 Würzburg, Germany; 3 Department of Spinal Surgery, The Second Hospital of Dalian Medical University, Dalian Medical University, Dalian 116023, China; 4 New Technologies Research Centre, University of West Bohemia, 30100 Pilsen, Czech Republic; 5 Department of Machining Technology, Faculty of Mechanical Engineering, University of West Bohemia, 30100 Pilsen, Czech Republic; 6 Department of Orthopedics and Trauma Surgery, Musculoskeletal University Centre Munich (MUM), Ludwig-Maximilians-University (LMU) Hospital, 80336 Munich, Germany; 7 Department of Conservative Dentistry and Periodontology, University Hospital Regensburg, 93055 Regensburg, Germany; 8 Department of Operative Dentistry and Periodontology, Center for Dental Medicine, Medical Center—University of Freiburg, Faculty of Medicine, University of Freiburg, 79106 Freiburg, Germany; 9 Department of Material Science and Metallurgy, University of West Bohemia, 30100 Pilsen, Czech Republic

**Keywords:** titanium scaffolds, laser surface texturing, implant integration, mesenchymal stromal cells, cell response, in vitro osteogenic differentiation, osteoporosis

## Abstract

The key to proper implant integration in bone replacement is to orchestrate the complex interactions between materials and tissues. Moreover, due to the rapid demographic shift towards aging societies and the increase in elderly and osteoporotic patients, it is of great importance that implant materials are osteointegrative in not only healthy but also compromised bone tissues. Here, titanium (Ti) scaffolds were subjected to shifted laser surface texturing (sLST) using a nanosecond pulsed laser to create an open pore macrotopography with micro-and nano-Ti droplets. In contrast to conventional laser texturing, which leads to high heat accumulation; in sLST, the frequency of laser pulses is low, allowing for resolidification, thereby creating a surface with abundant coverage micro-/nanodroplets. The main objective was to compare the cellular responses of human mesenchymal stromal cells (hMSCs) on sLST-textured Ti surfaces (LT-Ti) for the first time with standard sand-blasted, acid-etched surfaces (SLA-Ti). In-depth analyses of cell survival, proliferation, shape, mineralization, and gene expression were performed. Cell survival/proliferation was found to be similar on both surfaces; however, SEM imaging revealed differences in hMSC morphology. On LT-Ti, cells adopted well-rounded shapes, whereas on SLA-Ti they assumed more planar shapes. Bulk RNA sequencing performed after short-term culture on both surfaces disclosed expression changes in genes such as *DUSP6*, *TNFSF12-TNFSF13* and *SULT1A4*. Remarkably, the osteogenic differentiation capacity of hMSCs was significantly enhanced on LT-Ti compared to SLA-Ti. Furthermore, aged/osteoporotic donor cohorts showed significantly enhanced matrix mineralization on LT-Ti. In conclusion, our novel results demonstrate that sLST-Ti surfaces are safe, highly biocompatible, can rescue patient-cohort-specific mineralization behavior, and therefore hold great potential for the development into next-generation implants, which are suitable for both the elderly and bone-compromised populations.

## 1. Introduction

Intraosseous implants have a long history of development and successful use in patients. In 2020, over 0.25 million hip and knee implant surgeries were documented in Germany [1]. There has been a sustained effort over the decades in assessing and improving their material properties. However, implant failure due to a variety of reasons (e.g., patient-dependent response, early or late aseptic loosening, implant infection) remains an important clinical problem in modern orthopedics. In Germany, for example, aseptic loosening was one of the most common causes of implant revision in 2020, accounting for approximately 25% and 23% of hip and knee replacements, respectively [1,2]. Different steps have been taken pre-, intra-, and post-operatively to counteract this problem. One pre-operative optimization option is the choice of the most appropriate implant composition [3]. Over the years, titanium has become the gold standard in the field of cementless implantation in orthopedic surgeries. Titanium and its alloys have a low elastic modulus (ca. 55–110 GPa), which is closer to native bone (ca. 20 GPa) in comparison to other metal materials (orthopedic Co-Cr alloy: ca. 200–250 GPa), as well as a low corrosion rate and, most importantly, excellent biocompatibility [3,4]. Despite many inherent advantages of titanium, adequate surface conditioning is required to create a stable integration in the surrounding bone tissue and to prevent implant failure. Roughening of the surface is a well-known technique for improving implant integration in bone since it benefits pro-osteogenic cells, whereas it inhibits other cell types, such as pro-fibroblastic cells [5,6,7]. A widely used technique to create rough implant surfaces is sand-blasting and acid-etching (SLA) [8,9]. One of the modern ways of surface modification is laser surface texturing, which has gained more attention in recent years and has partly been commercialized for use in dental applications [10,11]. A special variant of this technology, with a shifted pulsed laser beam approach, is the sLST technique [12,13]. In comparison to existing classical methods of laser surface texturing, the sLST method has a special time–space distribution of laser spots [14]. In brief, the next laser pulse arrives on the same micro-object only after one complete scanning layer is finished. In classical methods, the frequency of laser pulses is up to MHz, leading to high heat accumulation. In contrast, the frequency of laser pulses in sLST is low because the period between pulses is equal to the duration of one scanning layer. Therefore, it allows for resolidification and oxidation between two laser pulses. The ablation caused by such deferment to later-stage laser pulses deposits oxidized layers on the textured surface in the form of irregular micro-/nanodroplets. Thereby, this laser technique can generate implants with predesigned macro-topographies and a high abundant coverage with micro-/nanodroplets. This can be a first step towards next-generation implants with patient-specific needs in terms of porosity and integrative capacity. Implant integration in the bone tissue occurs in consecutive stages: (i) initial integration via formation of woven bone around the material; (ii) adjustment of the bone mass in a load dependent manner via deposition of lamellar and parallel fibered bone; and (iii) load specific adaption by remodeling of bone tissue. Bone healing during implant integration is a complex, well-coordinated process that starts autonomously when the bone tissue is injured [15]. It begins with an inflammation phase (formation of hematoma), that attracts vascular and cells of the immune system. Subsequently, mesenchymal stromal cells (MSCs) that have progenitor characteristics migrate to the injury site and bone repair gradually advances, including differentiation of MSCs towards osteoblasts and osteocytes and deposition of bone-related matrix proteins such as type I collagen and osteocalcin. As MSCs are one of the first cells recruited to the bone–implant interface and play a significant role in bone tissue repair, their implementation in evaluating osteogenic capacities of implant surface treatments is of great importance [15,16]. In the case of osteoporosis, a bone disease characterized by mis-balanced bone turnover and reduced mineral density, research using animal models demonstrated multiple obstacles during bone repair, such as delayed cell differentiation, slow bone growth and poor mineralization [17]. Hence, considering the rapid demographic shift towards aging societies and the increase in elderly and osteoporotic patients, it is of great importance that the development of implant materials targets effective osteointegration in not only healthy but also in compromised bone tissues.

**Figure 1:**
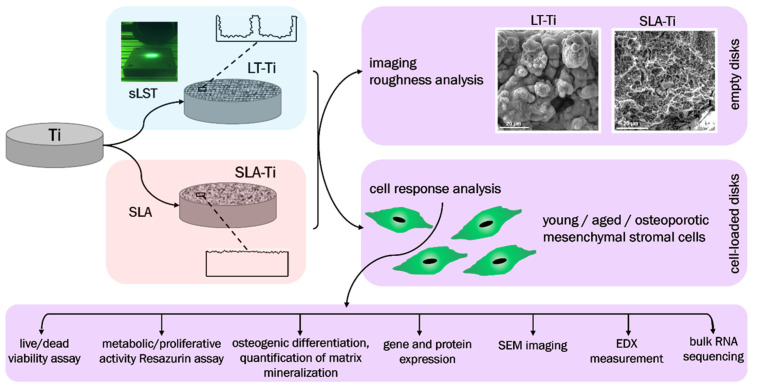
Schematic overview of the study workflow.

The major goals of this study were (i) to evaluate complex cellular responses towards sLST-generated laser-textured titanium (LT-Ti) surface with focus on primary human MSCs derived from young/healthy patients; (ii) to compare the effect on the osteogenic mineralization of three different cohorts: young/healthy, aged/healthy and aged/osteoporotic and (iii) by implementing RNA sequencing technology, to identify potential gene candidates involved in cell surface interaction. The main hypotheses were that the LT-Ti surface: (1) will better mimic, due to increased roughness and generation of micro/nano droplets, morphological features of natural bone tissue; (2) is biocompatible in terms of cell survival and proliferation; and (3) augments mineralization of three different hMSCs cohorts. SLA-treated Ti disks (gold standard in bone implants) and polystyrene (PS) culture dishes (standard surface used in vitro for cell cultivation) were used as control surfaces. The study workflow is shown in Figure 1, focusing on the comparison of LT-Ti to SLA-Ti and the performed analyses for empty and cell-loaded disks.

## 2. Materials and Methods

### 2.1. Titanium Disk Preparation and Characterization

Commercially pure titanium rods, referred to as titanium grade 2, (INKOSAS, Praha, Czech Republic) with a diameter of 12 mm were cut into disks with a height of 1.2 mm. The cut samples were first treated with sandpaper (400 grit). Next, disks were subjected to the shifted laser surface texturing method [18] using an SPI G3 series laser (beam wavelength 1064 nm, pulse duration 200 ns; TRUMPF, Ditzingen, Germany), as described previously [19,20]. As a control surface, disks were SLA-treated by sand-blasting (large grit, corundum sand with the main chemical compounds Al_2_O_3_ and SiO_2_) and acid-etching in 67% HCl/H2SO4 (1:1) at 80 °C for 10 min. The generated LT-Ti and SLA-Ti disks were subjected to SEM imaging (scanning electron microscope, acceleration voltage 15 kV; TESCAN, Dortmund, Germany). Surface topography and colored height scale images were taken with a digital Keyence VHX-6000 microscope (Keyence, Osaka, Japan). The instrument scanned the surface using a high-resolution optical sensor, capturing detailed 3D-topographical information. The collected data for surface roughness measurements was processed using Keyence CV-H1X Simulation-series software (Version 5.8). Roughness measurements of the disk surfaces (LT-Ti: *n* = 2, r = 9; SLA-Ti: *n* = 1, r = 9) were performed using a confocal microscope (405 nm violet laser, height display resolution 0.5 nm; OLS5000 SAF, Olympus, Tokyo, Japan) equipped with analysis packages for calculation of Rq and Ra values. Rq is the root mean square roughness, namely, the statistical average of the squared height deviations from the mean surface and represents the overall surface roughness, considering both peaks and valleys. Ra is the arithmetical average roughness and calculates the average absolute height deviations from the mean surface. XRD, FTIR, Raman and XP-spectroscopy have been described in detail by Křenek et al. [19]. These analyses validated surface oxidation and surface occurrence of predominant TiO (hongquiite) together with lower amounts of α-Ti phase, Ti_2_O_3_, Ti_3_O, anatase and rutile. In terms of wettability of LT-Ti surfaces treated by nanosecond lasers, this was studied in detail in previous publications, showing superhydrophilic and switchable wetting behavior [21,22].

### 2.2. Cell Culture

#### 2.2.1. Cell Types

hMSCs were isolated as described [23] from iliac crest bone marrow aspirates following informed patient consent approval by the local Ethical Committee of the Medical Faculty of the University of Regensburg, Germany (Ethic approval Nr. 00/134). Cells were expanded under standard cell culture conditions in DMEM basal medium (low glucose; Gibco, Darmstadt, Germany) supplemented with 10% fetal bovine serum (FBS; PAN Biotech, Aidenbach, Germany) and 1% Penicillin–Streptomycin (Pen–Strep; Sigma-Aldrich, Darmstadt, Germany). A cohort of hMSCs donors (young/healthy, YH-hMSCs, *n* = 10, Table 1) was characterized and validated for their progenitor cell characteristics. Based on their mineralization capacity during osteogenic differentiation (refer to results Section 3.3), six YH-hMSCs donors (*n* = 6) were selected for detailed cell response analyses on LT-Ti and SLA-Ti disks. Two additional cohorts, aged/health (AH-hMSCs) and aged/osteoporotic (OP-hMSCs), were generated under ethical grants Nr. 238-15 and Nr. 19-177 by the Ethical Committee of the Medical Faculty of the LMU, Munich, Germany. AH- and OP-hMSCs were isolated from the spongy bone of the femoral head and cultured as previously published [24,25]. Prior to surgery for the AH- and OP-cohorts, patients were examined for osteoporosis, including X-ray and DEXA (dual energy X-ray analysis), and thereby grouped. Cells were analyzed within passages 2–4. Furthermore, the previously established hMSC derivative cell line SCP-1 (hTERT immortalized, *n* = 1), described in [26], HUVEC-TERT cell line (*n* = 1; ATCC, Manassas, VA, USA) and THP-1 macrophage cell line (*n* = 1; ATCC, VA, USA) were used.

#### 2.2.2. Cell Culture on LT-Ti and SLA-Ti Disks

Primary hMSCs and SCP-1 cell line were cultured in proliferation medium consisting of DMEM (low glucose; Gibco), 10% FBS (PAN Biotech) and 1% Pen–Strep (Sigma-Aldrich). HUVECs (human umbilical vein endothelial cells) were cultured in HUVEC expansion media (ATCC) supplemented with 2% FBS, 5 ng/mL VEGF (vascular endothelial growth factor), 5 ng/mL EGF (endothelial growth factor) and 5 ng/mL bFGF (fibroblast growth factor; all ATCC). The THP-1 cell line was cultured in RPMI-1640 media supplemented with 10% FBS (ATCC, VA, USA) and 1% Pen–Strep in flasks for suspension cell culture. For THP-1 differentiation to adherent M0 macrophages, cells were seeded (5 × 10^5^ cells/mL) in T75-flasks and then incubated for 48 h in RPMI-1640, 10% heat-inactivated FBS and 60 ng/mL phorbol 12-myristate 13-acetate (PMA; Sigma-Aldrich). Following PMA treatment, the attached macrophages were cultured in RPMI-1640, 10% heat-inactivated FBS for 24 h before further use.

Disks were washed in Millipore water and steam sterilized at 121 °C prior to cell culture use. HMSCs/SCP-1 were seeded onto the disks based on established assay protocols as follows: 1 × 10^4^ cells/disk for Resazurin assay and live/dead staining and 8 × 10^3^ cells/disk for osteogenic differentiation. HUVECs were seeded for all assays at 12 × 10^4^ cells/disk for all assays. Macrophages were seeded onto disks at a concentration of 16 × 10^4^ cells/disk for all assays. All used cell seeding densities are based on established assay protocols and depending on the assays, different starting densities were needed. Cell-loaded disks were placed in a 24 well dish and cultured until different time points for follow-up analyses.

### 2.3. Validation of Stem Cell Characteristics

Validation was carried out according to the minimal criteria defined by Dominici et al. [27]: standard culturing on polystyrene for plastic adherence, stimulation and staining for trilineage differentiation potential and flow cytometry for CD surface marker expression.

#### 2.3.1. Flow Cytometry

YH-hMSCs (*n* = 6) were screened for the presence of surface markers using the BD Stemflow hMSC Analysis Kit (Cat. Nr. 562245, BD Biosciences, Heidelberg, Germany). First, monolayer cells were washed in phosphate buffered saline (PBS), detached with Accutase (Sigma-Aldrich) for 15 min at RT, centrifuged and resuspended in FACS (fluorescence activated cell sorting) buffer (1% FCS in PBS) at a concentration of 2–5 × 10^6^ cells/500 µL. Afterwards, aliquots of cells were incubated with mouse anti human antibodies against the positive markers CD73/90/105, negative markers HLA DR, CD11b/19/34/45 and the respective isotype IgG controls (measurement of unspecific background signal) for 30 min in the dark on ice, as per manufacturer instructions. Next, cells were washed twice and resuspended in 300 µL FACS buffer. For each donor, one unstained control was treated with the same incubation and washing steps. FACS analyses were performed using the flow cytometer instrument BD FACSCanto MT 2L Blue + Red (BD Biosciences) and forward (FSC) and side scatter (SSC) as well as the FITC, PE, PerCP-Cy5.5, APC emitted fluorescence were measured. Unstained control samples were used for the first measurement per donor group to perform gating based on particle size and granularity (FSC and SSC), thereby excluding dead cells, doublets or debris. Measurements were performed until approximately 17,000–18,000 events were recorded within the gated population, resulting in an average of 40,000–50,000 total events acquired per donor. Data (*n* = 6) was analyzed with the open-source software FCSalyzer (Version 0.9.18-alpha) [28] and expressed as percentage of positive/negative stained cells.

#### 2.3.2. Osteogenic Differentiation and Alizarin Red S Staining

hMSCs were seeded (8 × 10^3^ cells/disk, thus normalizing by same cell input) onto LT-Ti or SLA-Ti disks or in 24-well polystyrene dishes and split into stimulated (r = 2 independent disks/donor) and control (non-stimulated, r = 1) groups. In the stimulated group, cells were given osteogenic stimulation medium, composed of DMEM high glucose, 10% FBS, 1% penicillin/streptomycin and 100 nm dexamethasone, 10 mM beta-glycerophosphate and 50 µM L-ascorbic acid (Sigma-Aldrich). In the control group, cells were cultured in medium composed of DMEM high glucose, 10% FBS and 1% penicillin/streptomycin. Media was changed twice a week for 21 days. On day 21, cells were washed with 1×PBS buffer and fixed with 10% formaldehyde (Sigma-Aldrich) for 15 min at RT. After several washing steps, disks were stained with 40 mM Alizarin Red S (ARS) solution (pH = 4.25, Sigma-Aldrich) at RT for 30 min. Next, all disks were air-dried and afterwards incubated with 10% CH3COOH (Merck, Darmstadt, Germany) for 30 min on a shaker to extract the bound ARS dye. The extracted solution was then incubated at 85 °C for 10 min, cooled and then centrifuged at 20,000× *g* for 15 min. Following pH adjustment of the supernatant to 4.2–4.3 with 10% NH4OH (Merck), the optical density was measured at 405 nm (Genios FL Fluorescence Plate Reader, Tecan, Männedorf, Switzerland). ARS concentration in mM was calculated using a standard curve (ARS solution with serial dilution from 2 mM to 0.03 mM).

#### 2.3.3. Adipogenic and Chondrogenic Differentiation

Details on methods and results are given in the Supplementary Information.

### 2.4. Live/Dead Staining

To assess the cell viability, live/dead staining was performed with hMSCs (*n* = 6, r = 1 disk/donor) cultured on the LT-Ti and SLA-Ti disks for 5 days. Briefly, the cells were washed with 1×PBS and stained with 5 μg/mL Calcein AM (labeling live cells in green) and 5 μg/mL Ethidium homodimer-1 (labeling nuclei of dead cells in red) (Molecular probes, Eugene, OR, USA) at 37 °C for 60 min. After washing with PBS, fluorescence imaging was conducted with a AxioObserver 7 microscope (Carl Zeiss Microscopy, Jena, Germany) equipped with the microscope camera Axiocam 503 color (Zeiss, Oberkochen, Germany).

### 2.5. Resazurin Assay

To evaluate the metabolic/proliferative activity on both disk types, cells (YH-hMSCs *n* = 6, r = 3 disks/donor; OP-hMSCs *n* = 5, r = 3; AH-hMSCs: *n* = 5, r = 3; THP-1 macrophage cell line *n* = 1, r = 2) were seeded at a density of 1 × 10^4^ MSCs/disk while 16 × 10^4^ THP1/disk on day 0. Resazurin measurement was performed on day 1, 3 and 7. At each time point, the culture medium was supplemented with 10% (*v*/*v*) Resazurin solution (0.8 mM, Sigma-Aldrich) at 37 °C for 3 h. Afterwards, 100 µL of the supernatants were photometrically measured at 545 nm excitation and 590 nm emission filters using the fluorescence plate reader (Tecan, equipped with Magellan software, Version 6.6). For each study group, the results were expressed as fold change to day 1.

### 2.6. Immunocytochemistry

For immunocytochemical staining of THP-1 macrophages, please refer to Supporting Information Section S1.4.

### 2.7. Gene Expression Analysis

AH-/OP-hMSCs (*n* = 5) on PL were lysed with QIAzol Lysis Reagent (Qiagen, Hilden, Germany) and total RNA was extracted using the RNeasy Mini kit (Qiagen) according to the manufacturer’s instructions. First-strand cDNA synthesis was performed with the Transcriptor Kit (Roche, Karlsruhe, Germany) using a minimum of 0.3 µg total RNA per reaction. QRT-PCR reactions for human Collagen1A1 and housekeeping gene human GAPDH (both Eurofins, Ebersberg, Germany) were conducted with the primer sequences given in Table 2. For osteogenesis-related genes Runx2 and ALP, primers were purchased from BioRad Laboratories (sequences are not provided by the company) and used together with the SsoAdvanced Universal SYBR Green Supermix Kit according to the manufacturer’s manual (all BioRad Laboratories, Feldkirchen, Germany). QRT-PCR was performed in the CFX96 detection system and data was analyzed with the CFX Maestro Software (BioRad Laboratories, Version 3.1). Retrieved data was normalized to the housekeeping genes GAPDH or HPRT and expression was calculated compared to the respective control group using ΔΔCt and 2^(−ΔΔCt)^ method.

### 2.8. Bulk RNA Sequencing

SCP-1 cells (2 × 10^4^ cells/disk; cell seeding and RNA harvesting took place three independent times; hence, LT-Ti, *n* = 3, for each r = 16 disks; SLA-Ti, *n* = 3, for each r = 12) were cultured in normal culture media for 24 h and then lysed with Qiazol. For each independent RNA preparation on either disk type, cell lysates from disks (r = 16 LT-Ti or r = 12 SLA-Ti) were pooled. RNA samples were retrieved as described in 2.4.3 and sent to Eurofins Genomics Europe Sequencing (Konstanz, Germany; paid service), where quality control and RNA sequencing via Illumina were conducted. Normalized data was provided from Eurofins and was then subjected to the subsequent bioinformatic analysis using the software R studio v2023.06.2 + 561 with “Bioconductor 1.30.22” package (Posit PBC, Boston, MA, USA). By implementing packages “edgeR 3.42.4” and “limma 3.56.2”, the normalized counts data was transformed to a logarithmic scale for differential gene expression analysis between LT-Ti and SLA-Ti groups. Following, “AnnotationDbi 1.62.2” and “org.Hs.eg.db 3.17.0” packages were applied to map gene annotations in ENTREZID and SYMBO formats. The acquired results were input to “EnhancedVolcano 1.18.1” to generate a volcano plot that provides an overview of not only up- and downregulated genes but also show statistical significance. After filtering, differentially expressed genes (DEGs, the top 20) between LT-Ti and SLA-Ti groups were screened out by ranking |logFC| values from high to low and a clustered heatmap was plotted for illustration by using “pheatmap 1.0.12” package. Package “clusterProfiler” was administered to perform GO and KEGG enrichment analysis and to construct dot plots of associated results. The obtained sequenced data is property of the authors and will be published on the NCBI database after acceptance of the manuscript.

### 2.9. Osteocalcin Protein Analysis

YH-hMSCs (8 × 10^3^ cells/disk; *n* = 3 donors on each LT-Ti and SLA-Ti, for each r = 2 disks/donor) were cultured in osteogenic stimulation or control medium. Cell culture supernatants were collected over 21 days. Supernatants were then concentrated (3 kDa < samples < 50 kDa) by centrifugation using kDa-cut off columns (Osteocalcin protein: 5.6 kDa; Merck). Protein concentration was measured using a BCA assay kit (Merck). Osteocalcin concentration was evaluated versus standard curve calculations using the Human Osteocalcin SimpleStep ELISA Kit (Cat. Nr. ab270202, abcam, Cambridge, UK). Samples were diluted 1:10 with the provided diluent. The assay was performed in 96-well format and the optical density at 450 nm was measured with a plate reader equipped with Magellan software Version 6.6 (both Tecan).

### 2.10. Scanning Electron Microscope Imaging and EDX Analysis

YH-hMSCs seeded on LT-Ti and SLA-Ti disks were either cultured in normal medium (*n* = 3, r = 1 disk, 1 × 10^4^/disk) for 5 days or in osteogenic stimulation/control medium for 21 days (*n* = 3, r = 1 disk, 8 × 10^3^ cells/disk) for SEM imaging (both) and EDX analysis (osteogenic stimulated/control). Cells were rinsed with Sörensen’s Buffer (0.1 M, pH = 7.4), incubated in 2.5% glutaraldehyde (Serva Electrophoresis, Heidelberg, Germany) and rinsed twice with Sörensen’s buffer. Salts were removed by washing thrice in double distilled water (ddH_2_O) for 5 min. This was followed by a dehydration series with ethanol (solved in ddH_2_O), each step twice for 10 min (30%, 50%, 70%, 80%, 90%, 96%) and a final anhydrous 100% ethanol step. Critical point drying was performed in CO_2_ with the device Balzers CPD 030 (FL-9496; BALTIC-Präparation, Wetter, Germany). Next, samples were mounted on aluminum stubs with carbon adhesive tabs (12.5 mm Ø, BP 2152; BALTIC-Präparation) and coated with carbon using the sputter coating device Bal-tec SCD005 (BALTIC-Präparation; 50 mm working distance, vacuum 0.05 mbar; with carbon yarn evaporation additive CEA 035). For visualization of a control natural bone microstructure, bovine bone slices were purchased from Immunodiagnostic Systems Holdings PLC (Tyne and Wear, UK), which were previously also involved as a control in a study [29]. Prior to SEM imaging, the bone specimen was fractured into two semicircles, mounted on aluminum stubs using double-sided adhesive carbon disks and conductive adhesive paste (Leit-Tabs 12 mm Ø G 3347, BALTIC-Präparation), and sputter-coated (5 nm platinum thickness, BAL-TEC SCD 005, Balzers, Liechtenstein; platinum foil BP 2228, BALTIC-Präparation). The images of the surface were taken on a SEM (FEI Quanta 400 FEG, Thermo Fisher Scientific, FEI Deutschland GmbH, Frankfurt, Germany), in high vacuum mode using a backscatter electron (BSE) detector, acceleration voltage 10 kV, working distance 10 mm.

### 2.11. Relative EDX Analysis

EDX analysis for the relative deposition of calcium and phosphorus rich matrix components was performed on LT-Ti and SLA-Ti disks. Measurements took place after osteogenic stimulation/control culture for 21 days (YH-hMSCs, 8 × 10^3^ cells/disk). Three different random regions per disk were examined using the EDX analysis System: EDAX Octane Elect detector, APEX v2.0 (AMETEK EDAX), calibration with standard-less customized coefficients (SCC-factors), 50 µm aperture, 100 live seconds, amplification time 3.84 µs, image resolution 1024  ×  800 pixels. The elements analyzed were C, Na, P, N, Mg, Ca, O and Ti. Within each region, 6–9 spots or areas were measured for both the stimulated and control hMSCs. The data represents the atomic percentages of the deposited inorganic Calcium and Phosphorus components of the mineralized extracellular matrix. Exemplary elemental mappings were obtained by 200 µs dwell time, drift correction, amplitude time 1.92 µs and 400–500 frame iterations to collect necessary counts.

### 2.12. Statistical Analysis

Graph preparation and statistical calculations for all data, except of RNA sequencing results, were performed with GraphPad Prism 7 (San Diego, CA, USA). Data in graphs is either shown as a bar chart with mean and standard deviation or a boxplot with IQR (interquartile range; 25th to 75th percentile), median value and whiskers showing minimum and maximum value. ANOVA and unpaired *t*-test analyses were performed. The *p*-values *p* < 0.05 (*), *p* < 0.01 (**), *p* < 0.001 (***) and *p* < 0.0001 (****) were considered significant.

## 3. Results

### 3.1. LT-Ti Exhibited Comparable Morphological Surface Features at the Micro/Nano Level Comparable to Native Bone Tissue

**Figure 2:**
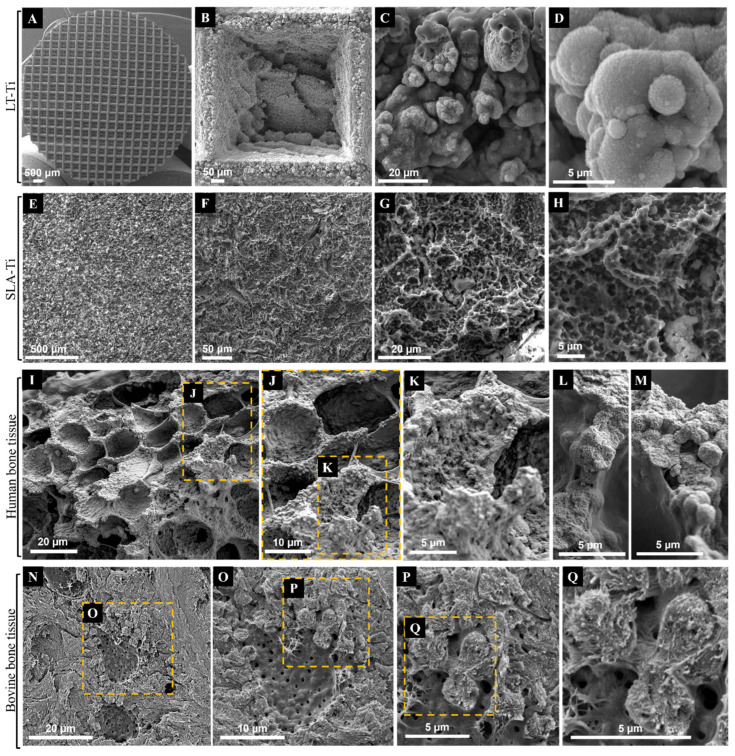
Representative SEM images of Ti disks after exposure to (**A**–**D**) shifted laser surface texturing, sLST or (**E**–**H**) sand-blasting and acid-etching (SLA) in comparison to representative SEM images of native (**I**–**M**) human bone tissue (purchased from Science Photo Library/Science Source/Nano Creative, www.sciencephoto.com) and (**N**–**Q**) bovine bone tissue. Magnifications are for (**B**): 253×, (**C**): 3 k×, (**D**): 11.2 k×, (**E**): 126×, (**F**): 646×, (**G**): 2.87 k×, (**H**): 6.19 k×, (**I**): 1.1 k×, (**L**,**M**): 4.4 k×, (**N**): 2 k×, (**O**): 4 k×, (**P**): 8 k× and (**Q**): 15 k×. Scale bars for (**I**–**M**) were calculated based on magnification related print size and pixel values.

SEM imaging was used to first evaluate the topography of the produced LT-Ti and SLA-Ti disks, and second, to compare them with each other as well as to natural bone tissue. The sLST technique generated a precise microgeometry of squared open pores (500 µm × 500 µm; 300 µm depth) (Figure 2A,B), a rough surface topography and a high coverage with irregular distributed micro- and nanodroplets (Figure 2C,D) below the range of 20 micrometers. SLA-Ti disks as well displayed a rough surface topography, although with rather sharp-edged cavities without nanodroplets (Figure 2E–H). In Figure 2I–L, representative SEM images of native bone tissue are shown. Structures observed on SLA-Ti (Figure 2G,H) were morphologically similar to those of natural bone tissue (Figure 2I,J), namely crater-like formations, which had smaller diameters than those in bone tissue. Such crater-like shapes are also visible on the macropore walls of the LT-Ti surface (Figure 2B). Interestingly, at high magnification, the micro- and nanodroplets of the LT-Ti surface (Figure 2C,D) presented great topographical parallels to features of human (Figure 2K–M) as well as to bovine bone tissue (Figure 2P,Q). In sum, SEM imaging demonstrated that sLST surface texturing can create topographies similar to natural bone tissue at different scales.

### 3.2. Topographical Characterization Revealed Higher Surface Roughness of LT-Ti Compared to SLA-Ti

**Figure 3:**
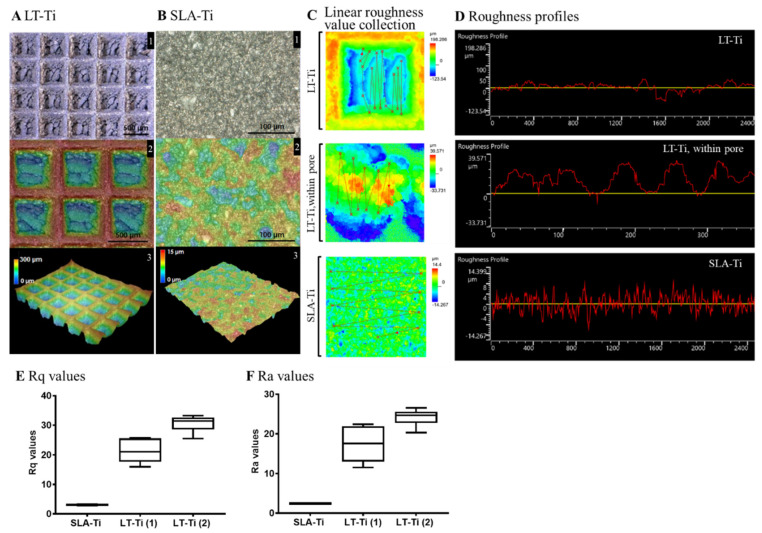
Topographical analysis of LT-Ti and SLA-Ti surface properties. Top views and height colored scale 3D-topographic images of (**A**) LT-Ti (blue: 0 µm, red: 300 µm) and (**B**) SLA-Ti (blue: 0 µm, red: 15 µm) disks. In A and B, the images indicated with 1, 2 and 3 show different magnifications, top and 3D-views. (**C**) Representative zoomed-in height colored scale images and scheme of roughness value collection, the lines connect the single measurement points. (**D**) Representative roughness profiles of LT-Ti (peaks to valleys deviating between ca. ±50 µm for LT-Ti pores) and SLA-Ti (peaks to valleys deviating between ±8 µm). (**E**,**F**) Rq and Ra values of LT- and SLA-Ti (*n* = 1 disk, boxplot and min/max; for LT-Ti(1) and SLA-Ti: r = 5 spots per disk; for LT-Ti(2): r = 6 spots; min/max for SLA-Ti too low to be visible). Rq (root mean square roughness) corresponds to the standard deviation of the height distribution within a measurement section. Ra values correspond to the average aberration of individual peaks and valleys from the arithmetic mean of the surface profile.

Surface roughness analysis of LT-Ti and SLA-Ti was conducted to quantitatively evaluate their topographical differences. Figure 3A displays the captured surface of LT-Ti disks in the XY-planes (A1, 2) and 3D images (A3) with the square pores of 500 µm × 500 µm and 300 µm depth. In contrast, SLA-Ti disks exhibited an abraded surface morphology without macro-pores (Figure 3B). Assessment of the roughness was performed by linear collection from 10 to 20 points in a defined area of 645 × 645 µm (for LT-Ti, this includes pore and edges) and for LT-Ti, an additional 129 × 129 µm (within the pore) (Figure 3C). Figure 3D shows the roughness profiles of LT-Ti (complete pore or within the pore) and SLA disk surface, representing a lateral view of the topography. Figure 3E,F show the mean Rq and Ra values of LT-Ti (1)/(2) (two independent measurements) and SLA-Ti. LT-Ti has seven times higher roughness values (in average 20–30) compared to SLA-Ti (<5). Altogether, the above data demonstrated that LT-Ti disks are characterized by several fold higher surface roughness (whole pore as well as within the pore) compared to SLA-Ti disks, which results in a higher surface area for the LT-Ti group that is also likely to impact cell and tissue responses.

### 3.3. Key Characteristics of YH-hMSCs Successfully Validated

**Figure 4:**
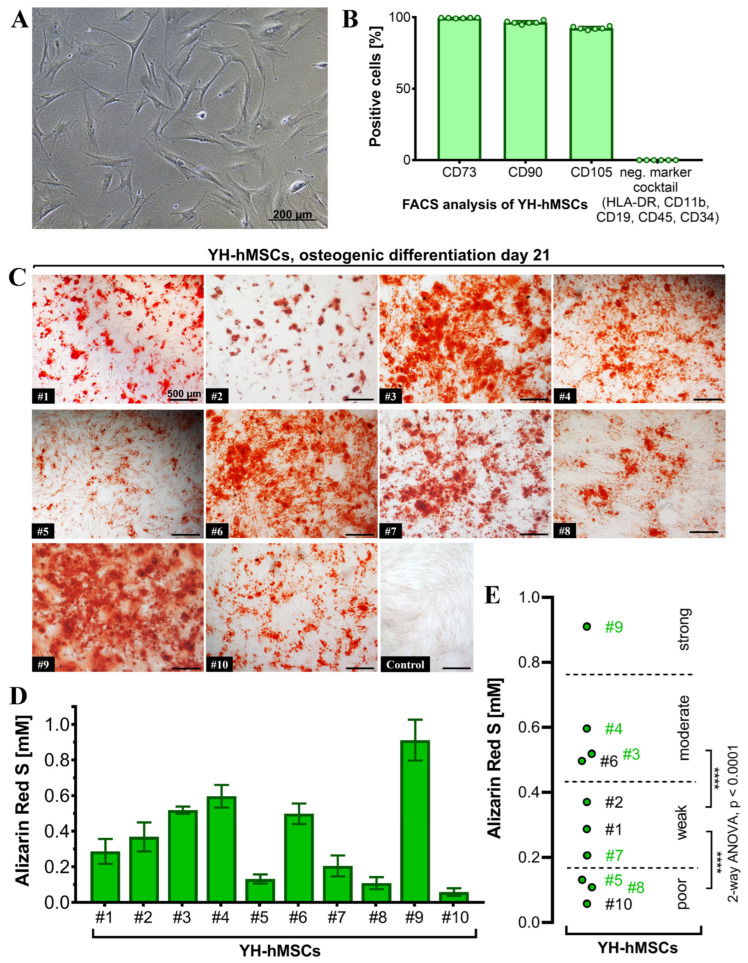
Characterization of the YH-hMSC cohort (*n* = 10, 5 female, 5 male; age 29 ± 6 years). (**A**) Representative phase contrast image of YH-hMSCs cultured in normal culture medium on polystyrene. (**B**) FACS analysis (*n* = 6) for positive (CD73: 99.57 ± 0.18%, CD90: 97.47 ± 1.1%, CD105: 92.46 ± 1.1%), and negative surface markers (HLA-DR, CD11b, CD34, CD45: 0.04 ± 0.02%). (**C**) Representative images for each donor of ARS staining on day 21 of osteogenic differentiation and one representative image after cultivation in control medium for 21 days and ARS staining. (**D**) Quantification of ARS (*n* = 10, r = 3 wells/donor; mean value ± SD). (**E**) Donor classification based on ARS quantification: poor (3 donors), weak (3 donors), moderate (3 donors) and strong (1 donor). Thresholds were chosen based on the highest significance (**** *p* < 0.0001) between groups when performing ANOVA. Donor numbers in green (*n* = 6; #3, #4, #5, #7, #8, #9) were included in the further experiments on LT-Ti and SLA-Ti.

All donors (*n* = 10) for the YH cohort were analyzed to validate their MSC characteristics and to also select 6 donors for follow-up culture on the LT-Ti and SLA-Ti. All YH-hMSC donors were adherent on polystyrene in standard cell culture conditions (representative image in Figure 4A). FACS analysis (*n* = 6) detected over 90% positive cells for the classical markers CD73 (99.57 ± 0.18%), CD90 (97.47 ± 1.1%) and CD105 (92.46 ± 1.1%), whilst the negative marker cocktail HLA-DR, CD11b, CD19, CD34 and CD45 showed negligible value of 0.04 ± 0.02% (Figure 4B). Representative scatter plots are shown in Figure S3 in Supporting Information.

Differentiation towards the three lineages was successfully validated for all donors with positive ARS staining indicating matrix mineralization for the osteogenic lineage (Figure 4C), metachromasia DMMB staining of sulfated glycosaminoglycans for the chondrogenic lineage (Figure S1), and Oil Red O visualizing lipid droplets for adipogenic lineage (Figure S2). The quantification of ARS revealed concentrations from 0.1 to 1 mM (Figure 4D). A statistical analysis of the initial 10 YH-hMSCs donors was performed and thresholds based on the calculated significant differences were set up to cluster their mineralization capacity into poor, weak, moderate and strong (Figure 4E). Afterwards, donors from each subgroup (2 poor: #5, #8; 1 weak: #7; 2 moderate: #3, #4; 1 strong: #9; labeled in green in Figure 4E), broadly covering donor variability, were selected for evaluation of the in vitro pro-osteogenic properties of LT-Ti and SLA-Ti. In conclusion, all YH-hMSC donors were successfully validated regarding the minimal criteria for hMSCs.

### 3.4. YH-hMSCs Cultured on LT-Ti Had Comparable Cell Survival and Metabolic/Proliferative Activity to SLA-Ti Controls

**Figure 5:**
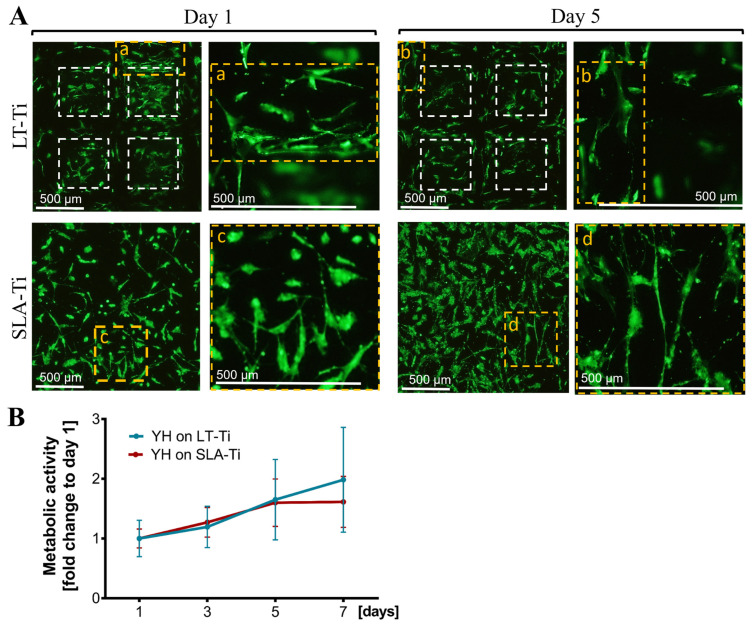
Cell survival and metabolic/proliferative activity. (**A**) Representative images of YH-hMSC cultured on LT-Ti and SLA-Ti at day 1 and 5, stained with Calcein AM (labeling living cells in green) and ethidium-homodimer1 (marking nuclei of dead cells in red). White dashed lines in LT-Ti images indicate the overlay of two z-level acquisitions—on the bottom and on the rim of the pore, while yellow dashed lines indicate regions that are magnified. (**B**) Resazurin assay for metabolic cell activity as a fold change to respective day 1 values (*n* = 6, r = 3 disks/donor, mean values ± SD). No significance was detected between YH-hMSCs on LT-Ti and SLA-Ti.

Next, a general biocompatibility assessment was carried out, comparing the novel LT-Ti surface to the standard SLA-Ti. Firstly, live/dead staining showed that hMSCs were able to adhere and spread on both disk types (Figure 5A, Day 1, representative images). The cells populated the edges and the bottom of the open pores of the LT-Ti group. By day 5, the majority of the hMSCs remained viable with only a neglectable number of dead cells detectable on both surfaces (Figure 5A, Day 5). Figure 5A demonstrates a top view of the cell bodies onto the LT-Ti and SLA-Ti surfaces and most of the cells had elongated bi- or tri-polar morphologies. A clear increase in YH-hMSCs metabolic/proliferative activity over time was detected on both disk types (Figure 5B). Altogether, the cell viability and metabolic/proliferative capacity were similar on both surface types.

### 3.5. LT-Ti Significantly Augmented the Osteogenic Differentiation of YH-hMSCs In Vitro

**Figure 6:**
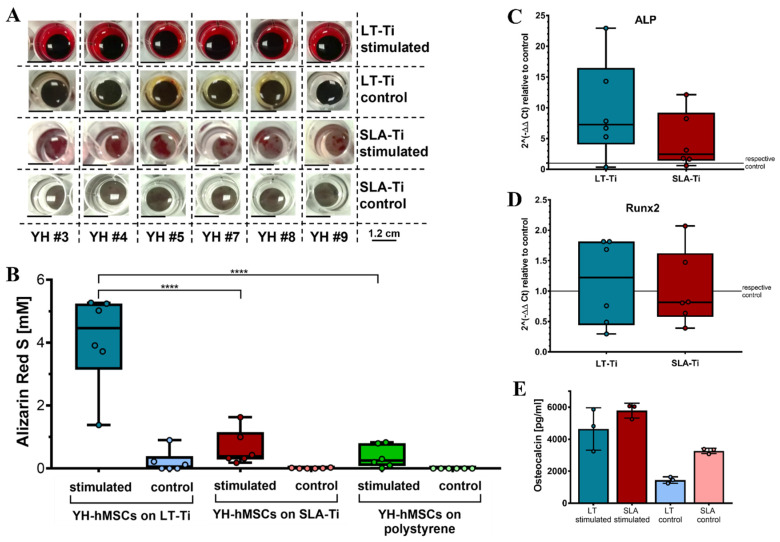
In vitro osteogenic differentiation of YH-hMSCs (*n* = 6, r = 3 disks/donor for stimulated, r = 2 for control; same for polystyrene condition) on LT-Ti and SLA-Ti. (**A**) Representative macroscopic images on day 21. (**B**) ARS quantification shown as boxplot (median ± IQR; whiskers indicating minimum and maximum; dots show single donors **** = *p* < 0.0001. Gene expression analysis data for ALP (**C**) and Runx2 (**D**) of YH-hMSCs on LT-Ti or SLA-Ti after 21 days of osteogenic stimulation. Fold changes were calculated versus their corresponding control. No significant differences between LT-Ti and SLA-Ti were detected for any of the genes. (**E**) Osteocalcin ELISA of YH-hMSCs cultured on LT-Ti and SLA-Ti in stimulated and control condition (*n* = 3, r = 2 measurements/donor; mean values ± SD; dots show single donors). Significances were detected between stimulated and respective control groups (LT-Ti: **, SLA-Ti: *). No significance was detected between LT-Ti stimulated and SLA-Ti stimulated.

To assess whether LT-Ti surfaces can lead to enhancement of osteogenesis in vitro, osteogenic stimulation and evaluation of YH-hMSCs were performed. Deposition of mineralized matrix, visualized by ARS staining, was observed on each of the culture surfaces (Figure 6A). SLA-Ti showed enhanced staining compared to polystyrene, however, LT-Ti surface exhibited the clearest enrichment of staining. This was validated by ARS quantification, revealing a highly significant 6-fold increase in ARS concentration in the LT-Ti group compared to the SLA-Ti group (****, *p* < 0.0001; Figure 6B). Comparison of individual donor behavior clearly demonstrated that poor and weak donors benefit tremendously from LT-Ti surface (Figure S4). Gene expression analysis comparing YH-hMSCs on LT-Ti or SLA-Ti after 21 days of stimulation to their corresponding controls, revealed a tendency towards increased levels of ALP and to a lower extent of Runx2 for LT-Ti (Figure 6C,D). To further confirm the osteogenic differentiation, ELISA analysis for a well-known bone protein marker, osteocalcin, was carried out with cells cultivated on both disk types. Higher osteocalcin levels in the cell culture supernatant of stimulated cells versus respective controls (Figure 6E) were determined. In the stimulated groups, no significant difference was detected in osteocalcin concentration between LT-Ti and SLA-Ti. In sum, LT-Ti significantly augmented the in vitro osteogenic differentiation of YH-hMSCs and thus, outperformed the SLA standard implant surface.

### 3.6. SEM/EDX-Analyses Independently Confirmed Matrix Mineralization on LT-Ti and SLA-Ti

**Figure 7:**
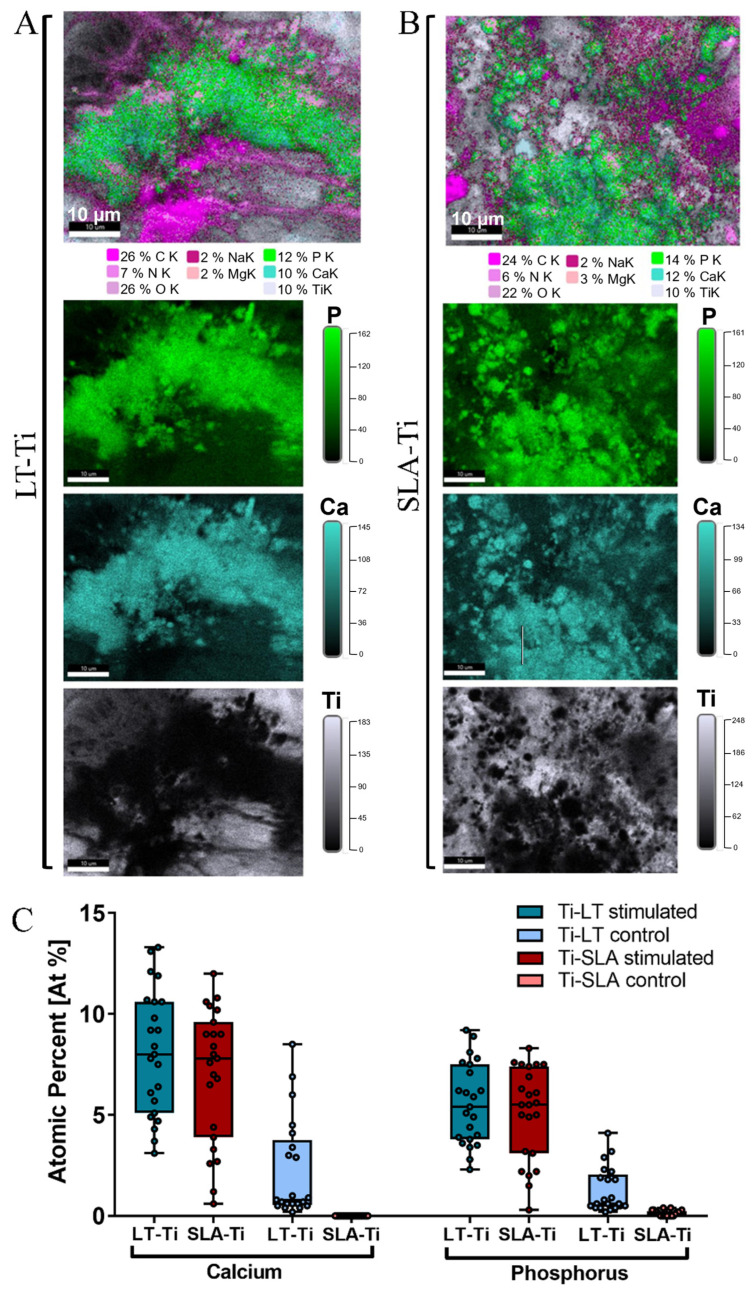
EDX elemental mapping of osteogenically stimulated and control YH-hMSCs (*n* = 1) on LT-Ti and SLA-Ti disks. Representative images of the elemental composition on (**A**) LT-Ti stimulated and (**B**) SLA-Ti stimulated groups. Top images are merged, while the three images below are extracted to show individual elements (P, Ca, Ti). (**C**) Quantification of the atomic percent (At%) of calcium and phosphorus for YH-hMSC-loaded LT-Ti and SLA-Ti disks in stimulated and control conditions. Data is presented as boxplot (median + IQR; whiskers indicating minimum and maximum; dots show individual spot measurement, r = 21–25).

To validate the deposition of minerals, SEM/BSE (backscattered electrons) imaging and EDX elemental mapping were conducted with YH-hMSCs differentiated on LT-Ti and SLA-Ti disks for 21 days. Within the LT-Ti pores, a dense layer of cell bodies and matrix is displayed (Figure S5A), as well as on the SLA-Ti surface (Figure S5B). The presence of mineral deposits in the matrix was evident in both groups (Figure S5, yellow arrows). EDX mapping and quantification of the atomic composition of different elements (Figure 7A,B) revealed high intensity signals for calcium and phosphorous, indicating matrix mineralization. Moreover, an increased atomic percentage of calcium and phosphorus was detected on both LT- and SLA-Ti compared to control groups (Figure 7C). Interestingly, for the LT-Ti group, small amounts of calcium and phosphorus were also found even in the controls. Next, the Ca/P ratios were calculated and the mean values are 1.45 for LT-Ti stimulated and 1.48 for LT-Ti unstimulated, while 1.38 for SLA stimulated and 0 for SLA-Ti unstimulated. Taken together, the SEM/EDX methodologies independently confirmed the matrix mineralization on both disk types. However, due to regional spot analyses of the surfaces, the data does not have the power to capture and represent the global difference between mineralized matrix composition on LT-Ti and SLA-Ti, as shown in Figure 6B.

### 3.7. SEM Analysis of YH-hMSCs Cultivated on LT-Ti and SLA-Ti

**Figure 8:**
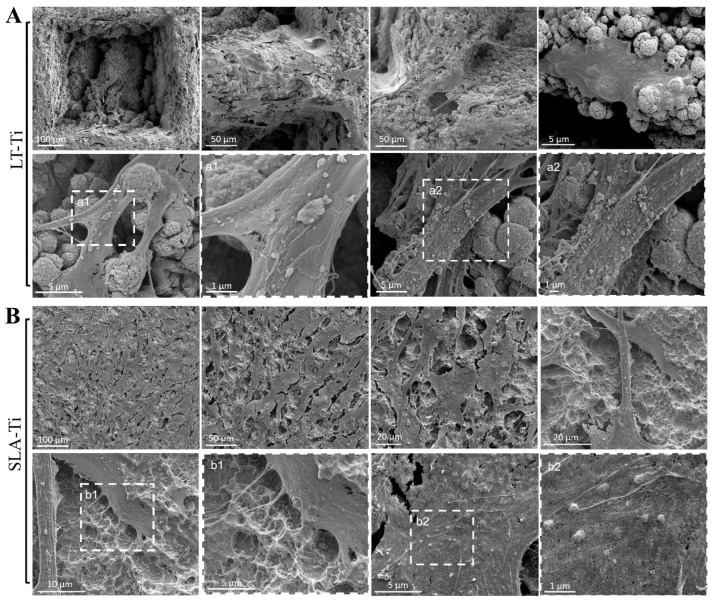
Representative SEM images of YH-hMSCs (*n* = 3) cultured on (**A**) LT-Ti and (**B**) SLA-Ti for 5 days. A magnification of the rectangulars a1/a2 and b1/b2, framed with white dashes, is is shown for each in the image to their right.

Based on Calcein AM labeling (Figure 4A), the top view of cell morphology was monitored and, on both, LT-Ti and SLA-Ti YH-hMSCs adopted bi- or tri-polar shapes. Next, SEM analysis was performed to monitor cells to material interface at a higher resolution (Figure 8). YH-hMSCs on LT-Ti (Figure 8A) covered the macropore edges, walls and bottom of the disk and exhibited cell bodies that followed the curvature of the micro-/nano granular surface. On SLA-Ti (Figure 8B), cells formed a monolayer and extended over the edges of the crater-like shaped surface with a rather planar appearance. Hence, it will be of interest to further characterize the three-dimensional cell morphology and whether the different surface topographies influence adhesion- and cytoskeleton-related signaling.

### 3.8. RNA Sequencing of hMSC Cell Line, SCP-1 Pointed Out Intriguing Trends in Differential Gene Expression

**Figure 9:**
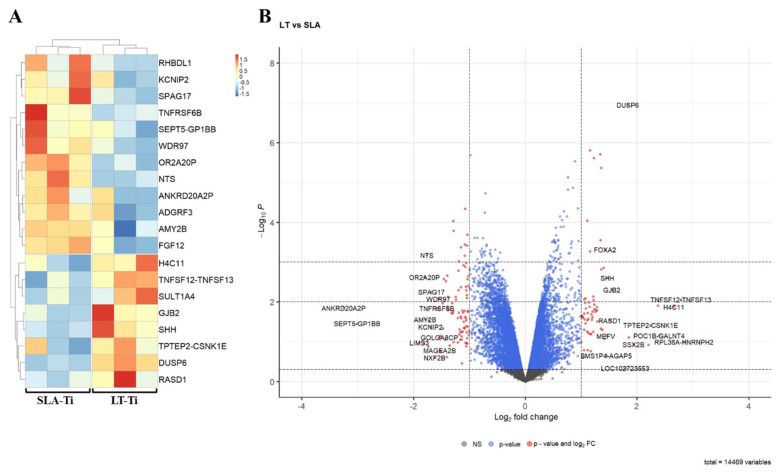
Bioinformatic analysis of RNA sequencing data of hMSC cell line, SPC-1 (*n* = 1). (**A**) Heatmap of the top 20 differentially expressed genes between SLA- Ti and LT-Ti (each *n* = 3 independent RNA preparations/group). Red on the color key indicates upregulation, while blue downregulation. (**B**) Volcano plot with vertical threshold at |Log2| = 1 (red dots). Top 30 genes (*p* < 0.05) are labeled with their SYMBOL IDs, respectively.

To capture very early molecular response towards the two different surface topographies, bulk RNA sequencing-based gene expression profiling was performed. For this reason, SCP-1 cells were given only 24 h to perceive the surfaces. Bioinformatic analyses of RNA sequencing data exposed only a small number of DEGs (differentially expressed gene) in SCP-1 cells when cultured for this brief period on LT-Ti and SLA-Ti. Figure 9A depicts a heatmap of the top 20 DEGs between LT- and SLA-Ti groups. In Table 3, the top 20 DEGs are listed with their Log2 FC values, *p*-values, full names and associated function, showing eight upregulated genes (e.g., *TNFSF12-TNFSF13*, *H4C11*, *SULT1A4*, *DUSP6*, *GJB2*, *RASD*, *SHH*) and 12 downregulated genes (e.g., *FGF12*, *WDR97*, *RHBDL1*, *TNFRSF6B SPAG17*, *KCNIP2*, *ADGRF3*, *NTS*, *OR2A20P*, *SEPT5-GP1BB*). Firstly, to screen for a wide range of gene expression changes, less stringent statistical significance analysis was applied, based only on *p*-value < 0.05, with the notion that some gene hits are potentially false positives. The resulting volcano plot (Figure 9B) shows an overview of all genes analyzed (14,469) specifying the genes below −1 and over +1 at the *x*-axis (Log2 FC) with red-colored dots, marking genes that are responding to the LT-Ti topography. GO analysis of the DEGs showed enrichment of gene clusters with the top accumulated terms for biological process: “ERK1 and ERK2 cascade”, “response to lipopolysaccharide” and “leukocyte proliferation”, while for molecular function: “receptor ligand activity”, “signaling receptor activator activity”, “cytokine activity” and “cytokine receptor activity” (Figure S6). Subjecting all DEGs to KEGG signaling pathway analysis, “cytokine–cytokine receptor interaction” was uncovered with the highest output, followed by “MAPK signaling pathway”, “viral protein interaction with cytokine and cytokine receptor” and “NF-kappa B signaling pathway” (Figure S7). A more stringent analysis was carried out by applying |LogFC| ≥ 1 and adjusted *p*-value < 0.05 and only five DEGs were fished out (Supplementary Table S1). This new data suggests that the LT-Ti disk topography induces specific, early responder genes in hMSCs that might be of interest for further investigation on surface-dependent orchestration of molecular signaling.

### 3.9. LT-Ti Significantly Enhanced the Osteogenic Capacity of Aged and Osteoporotic hMSCs

**Figure 10:**
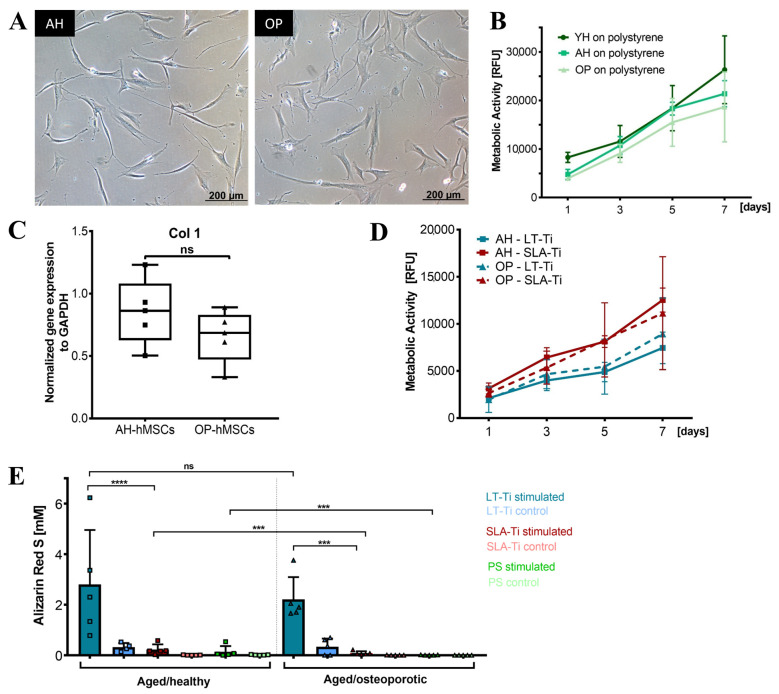
Response of aged/healthy (AH) and aged/osteoporotic (OP) hMSCs to LT-Ti and SLA-Ti disks. (**A**) Representative phase contrast images of AH- and OP-hMSCs. (**B**) Cell metabolic/proliferative activity on polystyrene (PS) in comparison to YH-hMSCs (AH and OP: *n* = 5, YH: *n* = 6). (**C**) COL1A1 gene expression of AH- and OP-hMSCs (*n* = 5, r = 4 disks/donor), normalized to GAPDH expression (ΔΔCq). Data is presented as a boxplot with donor values and median + IQR; whiskers indicating minimum and maximum. (**D**) Metabolic/proliferative activity for AH- and OP-hMSCs cultivated on LT-Ti and SLA-Ti surfaces (*n* = 5 donors, r = 3 disks/donor). Data expressed as mean values ± SD. (**E**) ARS quantification at day 21 of osteogenic stimulation of AH- and OP-hMSCs (*n* = 5) cultivated on LT-Ti, SLA-Ti and (PS). Data represents mean values + SD; *p* < 0.0001= ****; OP-hMSCs: *p* < 0.001 = ***, no signficance deteced = ns.

Based on the beneficial effects of LT-Ti on YH-hMSCs, cohorts from aged/healthy (AH-hMSCs) and aged/osteoporotic donors (OP-hMSCs) were included to assess if LT-Ti can also augment the in vitro osteogenesis of such cells. In 2D culture on polystyrene, the cell morphology of AH- and OP-hMSCs was similar to YH-hMSCs (Figure 3A and Figure 10A). Metabolic/proliferative activity of AH- and OP-donors over time was decreased compared to YH-hMSCs, with the lowest values being for OP-hMSCs (Figure 10B). Analysis of Collagen1 (COL1A1) gene expression indicated a tendency of lower COL1A1 mRNA levels in OP-hMSCs versus AH-hMSCs (Figure 10C). On LT-Ti and SLA-Ti disks, cells of both cohorts propagated over time and no significant differences were detected (Figure 10D). However, ARS quantification on day 21 of stimulation revealed a significant 10-fold and 24-fold increase in mineralization for AH- and OP-hMSCs respectively, when cultured on LT-Ti disks compared to SLA-Ti group. (Figure 10E, *p* < 0.001 = *** for OP-hMSCs, *p* < 0.0001 = **** for AH-hMSCs). Graphs separated to LT-Ti, SLA-Ti and PL groups can be found in Figure S8, as well as representative macroscopic images of ARS staining on LT- Ti and SLA-Ti. Taken together, the above data convincingly showed that LT-Ti surface also exerts significant beneficial effects on the osteogenic differentiation of cells derived from compromised bone tissues.

### 3.10. Behavior of HUVECs and THP-1 Macrophages on LT-Ti and SLA-Ti

HUVEC cell line was found to be viable on both Ti surfaces, although more dead cells were detectable on SLA-Ti surfaces (Supplementary Figure S9A). Resazurin assay demonstrated a 3-fold increase in metabolic/proliferative activity on LT-Ti versus SLA-Ti at day 5 of culture. Moreover, HUVECs had no apparent cell growth on the SLA-Ti disks in the studied time course (Figure S9B). CD31 expression was detected for HUVECs cultured on both disk types, but an early tubulogenesis was observed only within the pores of LT-Ti disks (Figure S9C).

**Figure 11:**
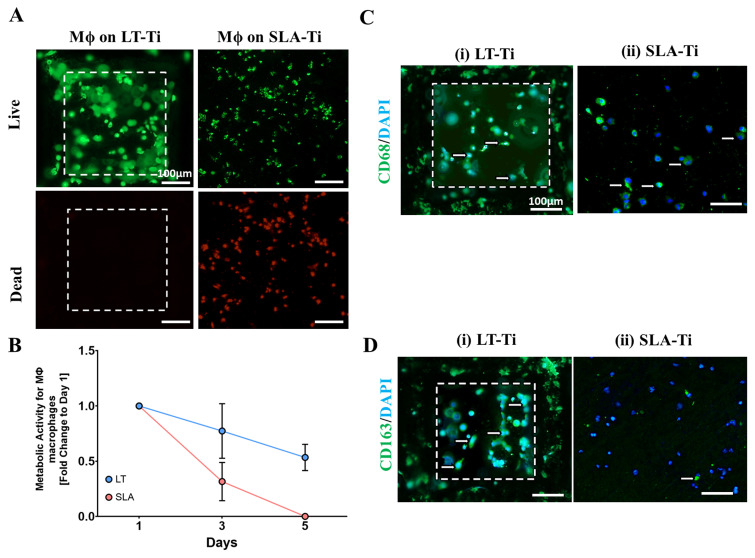
Response of adherent THP-1 macrophage cell line (*n* = 1) to LT-Ti and SLA-Ti disks. (**A**) Representative images of live (green) and dead (red) staining of macrophages (Mϕ) after 5 days culture on LT-Ti or SLA-Ti disks (r = 1 disk/group). (**B**) Metabolic/proliferative activity of macrophages on LT-Ti and SLA-Ti disks (mean + SD of r = 2 disks/group, *n* = 1). Representative images of (**C**) CD68 and (**D**) CD163 staining and DAPI counterstaining of macrophages on LT-Ti and SLA-Ti disks (r = 1 disk/group). White arrows indicate positive stained cells. White dashed lines in LT-Ti images indicate the overlay of two z-level acquisitions—on the bottom and on the rim of the pore.

Macrophages demonstrated greater viability on LT-Ti compared to SLA-Ti disks, which had more dead cells (Figure 11A). Resazurin assay correlated with these results, as macrophages on LT-Ti disks had 7-fold higher metabolic activity at day 5 compared to SLA-Ti disks, confirming that LT-Ti disks are more biocompatible for macrophages (Figure 11B). Staining for macrophage polarization markers showed that CD163-positive, or M2 macrophages, were predominant on LT-Ti disks, whilst the presence of CD68-positive, or M1 macrophages, were observed on SLA-Ti disks (Figure 11C,D). In sum, the analyses of vascular and macrophage cell types that are also involved in osteogenic process and biomaterial responses, demonstrated the benefetial effects of LT-Ti disks in terms of cell propagation, survival and reparative phenotype.

## 4. Discussion

In this study, LT-Ti was created using shifted laser surface texturing, sLST, technology with the aim of developing next-generation implants that guarantee enhanced osteointegration in elderly patients with compromised bone tissue quality. Despite the excellent clinical results of orthopedic implants, implant failure and revision risk are still major problems in modern medicine. Data from the German Endoprosthesis Register (EPRD) show a significant increase in revisions of primary uncemented prostheses after several weeks to months [1]. In a statistical survey, covering countries of Europe, as well as Australia and USA, the ten-year revision risk rates for total knee arthroplasties are 25% for uncemented and 35.7% for cemented procedures [35]. Moreover, for aged and especially for osteoporotic patients, cementless fixation of implants cannot be applied, due to the reduced bone density. In these cases, to ensure stable fixation, the cementing approach needs to be used, which is frequently an extensive procedure, as well as an aggressive intervention to the surrounding bone tissue. To further improve the longevity of implants, but also to enable uncemented arthroplasties with robust osteointegration in elderly and osteoporotic patient cohorts, the novel sLST technology was applied. Here, as an initial step towards next-generation implant development, the complex cellular response of bone forming, human mesenchymal stromal cells (hMSCs) on LT-Ti surface and gold standard, SLA-Ti surface, was studied and compared in great detail. The main findings were (i) sLST-Ti treatment produced a topography with a high degree of roughness and heterogeneity with stable micro- and nanodroplets, a surface that is similar to that of natural bone tissue at the microscopic level; (ii) the overall biocompatibility of LT-Ti and SLA-Ti surfaces was very comparable; (iii) SEM analysis revealed differing hMSCs in 3D, with bulk RNA sequencing data suggesting a distinct molecular response in the very early events of cell contact with the two surface types; and (iv) most importantly, LT-Ti surface induced a highly significant increase in matrix mineralization not only of young/healthy (YH) hMSCs but also of aged/aged hMSCs; and (iv) most importantly, the LT-Ti surface induced a highly significant increase in matrix mineralization not only of young/healthy (YH-hMSCs) but also of aged/healthy and aged/osteoporotic (AH- and OP-hMSCs) hMSCs, demonstrating the suitability of the LT-Ti material for the development of cementless implants for different patient cohorts. There have already been first steps taken towards the commercial use of laser-textured implants for dental applications [10,11], which strengthens the notion that laser texturing may lead to next-generation orthopedic implants. In this study, it was of foremost importance to initially compare the sLST-generated LT-Ti surface to the standard SLA that is widely used in medical applications. Our results demonstrated that the LT-Ti surfaces result in significant augmentation of mineralization not only by healthy but also diseased cells. With regards to mechanisms of action, additional controls such as the plasma sprayed Ti will be in the scope of follow-up research.

After the production of LT-Ti and SLA-Ti disks, their biocompatibility was initially analyzed. The assays “Live/Dead” (cell survival) and “Resazurin” (metabolic/proliferative activity) were used to evaluate the biocompatibility of the surfaces, as these assays are directly indicative of material cytotoxicity. No cytotoxic effect could be detected on the cell types assessed here, apart from THP-1 macrophages cultivated onto SLA-Ti disks. With regards to effects on vital organs that are distant from the bone tissue, such as heart, liver, and lung, this is within the scope of our follow-up in vivo study, where we will also evaluate if titanium nanoparticle released from the implant surface accumulates in vital organs. Next, their topographical properties were compared to one another and to native bone tissue. Native human bone, as well as bovine bone tissue, which is used as an alloplastic bone graft material in orthodontics for guided tissue regeneration [36] showed great similarity to LT-Ti with its irregular terrain containing stochastically distributed micro- and nanodroplets of titanium. These were formed via laser ablation of titanium and subsequent re-deposition of titanium species oxidized on the air and consist of titanium oxides and suboxides, as shown in a previous study by Křenek et al. (2021) [19]. In contrast, SLA-Ti had a sharp-edged and crater-like surface. In line with the structural data, Ra and Rq measurements demonstrated several fold higher roughness values for LT-Ti versus SLA-Ti, whilst the obtained SLA values were comparable to previous literature [37,38,39]. Wennerberg et al. [40] described a roughness range of 1–2 µm as appropriate for osteogenic purposes. However, with the progress of technology, multifold higher surface roughness can be now achieved, and it is of great importance to assess biological response and pave the way for further augmentation of biomaterials. Our novel data shows a significant enhancement of in vitro osteogenesis on LT-Ti disks that have higher roughness compared to the current medical SLA-Ti standard. Therefore, sLST-driven LT-Ti open pore morphology with nanodropletting may be the foundation of a novel cementless implant approach for patients with compromised bone quality, which is not an existing option so far. Interestingly, SEM imaging exposed different hMSC morphologies on LT-Ti, where the cells followed the micro- and nanodroplet curvatures and resembled mostly rounded three-dimensional shapes, while on the SLA-Ti, hMSCs had stretched, planar cell bodies. It has been previously reported that micro-/nanostructuring of materials can control cell adhesion and spreading, and in turn can affect MSC behavior including lineage differentiation [6,41,42,43,44]. For example, a surface with microgrooves and pillars below 10 μm leads to increased cell area and focal adhesion complexes, leading to enhanced MSC osteogenesis [45]. Studies have already demonstrated that cell shape can be a leading cue for osteogenic differentiation and there have been efforts to predict MSC osteogenic potential via morphological analysis [46,47]. Zhao et al. (2019) [46] assessed the effect of adhesive substrates arranged in different geometric forms and sizes and discovered a strong correlation between high spreading area and enhancement in MSC osteogenesis. In a further study, we will carefully investigate the three-dimensional shape of the cells and the relationship to mineralization and osteogenic processes. Moreover, in the current study, the initial cell number was normalized by starting with the same cell input to allow for direct comparison of cell behavior onto the different disk types. However, as our novel data showed that LT-Ti surfaces are much rougher than SLA-Ti in the follow-up investigations, normalization can be performed by cell density (cells/micrometer). Additionally, an in-depth exploration of the molecular mechanisms will be needed in the future to better understand the cellular impact of the material surface properties.

As a pilot approach to identify differential molecular response in hMSC towards LT-Ti and SLA-Ti and to have an unbiased view on the transcriptome response at a very early time point, bulk RNA sequencing of derivative SCP-1 cell line was conducted. Cells were given only 24 h for interaction with LT-Ti and SLA-Ti disks, to monitor very naive reactions to the surfaces and to avoid masking by serum-derived or autocrine extracellular matrix. Not surprisingly, at this time point, a small number of differentially expressed genes were detected. Therefore, a less stringent approach was applied by using a combination of log-fold-change and *p*-value, which still gives a reasonable first screening of the molecular shift between cells loaded onto LT-Ti or SLA-Ti. The more stringent approach based on adjusted *p*-value, which includes the rate of possible false positive hits into the calculations [48], was also given in the Supplementary Information. Some of the detected genes in the top 20 gene list, are referred to as pseudogenes [49] which are not prone to code for a functional protein and were most likely quietened during evolutionary developments. Interestingly, already at this early time for cell-surface contact, several genes responded to the two disk types. For example, *DUSP6* (Dual Specificity Phosphatase 6, *SULT1A4* (Sulfotransferase Family 1A Member 4) and *TNFSF12-TNFSF13* (tumor necrosis factor ligand superfamily member) genes were upregulated in SCP-1 cultivated onto LT-Ti. *DUSP6* encodes for a phosphatase involved in the regulation of MAPK superfamily (RAS–ERK–MAPK pathway) which subsequently affects cell proliferation and differentiation [50,51,52]. In recent research on osteoporosis, *DUSP6* was found to be downregulated in osteoporotic bone samples with its inhibition leading to an increase in osteoclast differentiation in vitro, while its upregulation had suppressive effect on osteoclasts [32]. Hence, the identified *DUSP6* mRNA upregulation on the LT-Ti surfaces can exert a beneficial effect on osteogenesis and counteract osteoporotic processes. *SULT1A4* is involved in catecholamine metabolism, as a catalysator of sulfate conjugation of hormones [31]. A downstream effector of *SULT1A4* is DHEA (Dehydroepiandrosterone), a frequent circulating human steroid hormone [53,54]. As reviewed by Kirby et al. (2020), clinical osteoporotic studies reported an increased bone mineral density after DHEA supplement treatment of elderly females [55]. At the cellular level, DHEA can directly influence osteoblasts and osteoclasts via estradiol and DHT (dihydrotestosterone) and osteoprotegerin expression, respectively [55]. DHEA addition to MSCs cell culture medium was described to stimulate osteoblast differentiation [55,56,57]. Thus, *SULT1A4* signaling could have, via DHEA, an impact on osteoblastogenesis of hMSCs exposed to LT-Ti surfaces. *TNFSF12-TNFSF13* is known to encode for proteins regulating cell apoptosis and found to act in osteoclastogenic pathways [58,59], thus it may play a role in bone remodeling at the implant site. In sum, even with the small number of responding genes at the 24 h of cell–material contact, it will be of great importance in follow-up studies to validate the changes by quantitative PCR as well as at the protein level, and to investigate the transcriptome at later time points and after osteogenic stimulation. Such analyses can result in mapping key molecular networks, orchestrating the intimate response of cells towards different surface topographies.

Next, in the process of novel cementless implant generation, it was of critical importance to evaluate the pro-osteogenic activity of the sLST surfaces. Our study clearly showed, by ARS staining and quantification, a highly significant increase in matrix mineralization when hMSCs interact with LT-Ti surface compared to the gold standard, SLA-Ti, and polystyrene control. SEM/EDX analysis of the elements, calcium and phosphorus confirmed their enrichment on both LT-Ti and SLA-Ti. Calculating the Ca/P ratio, which is a widely used parameter for characterizing mineral deposits, the stimulated samples reached mean values of 1.45 for LT-Ti and 1.38 for SLA-Ti. Interestingly, in the case of unstimulated samples, the mean value of Ca/P ratio was 1.48 for LT-Ti versus 0 for SLA-Ti, thus demonstrating that on the LT-Ti surface even without chemical stimulation, there is accumulation of mineral deposits. In human bone, the ratio ranges between 0.98 and 2.85, with a mean of 2.17 in healthy people [60]. Despite there being no perceptible, significant difference between LT- and SLA-Ti, this relates to the method which permits only sub-regional, and not global examination of the surfaces. Intriguingly, within the control groups, both calcium and phosphorus were detected exclusively onto LT-Ti, a phenomenon due to possible spontaneous osteogenic differentiation, which was also recognized in the ARS staining. This finding is in line with recent studies focusing on creating scaffold topographies for induction of spontaneous osteogenic differentiation without addition of pro-osteoblastic chemical stimuli in the culture media [61,62]. Another indispensable step in the successful peri-implant bone healing and thereby integration is appropriate tissue vascularization, necessary for the oxygenation and nutrition of the de novo laid bone. To confirm LT-Ti compatibility with endothelial cells, the response of a HUVEC cell line was evaluated and the data demonstrated that LT-Ti was superior to SLA-Ti, whereby the latter exhibited much lower HUVEC metabolic/proliferative activity in concomitance with higher incidence of PI-positive nuclei. Moreover, on LT-Ti surface, even an initial tubulogenesis-like event could be observed, thus strongly suggesting that vessel formation is facilitated by the sLST material. Additionally, a favorable immune response is necessary to enable bone–implant integration. Therefore, a subtype of immune cells, macrophages were cultured and examined on the LT-Ti and SLA-Ti disks. Interestingly, macrophages showed even higher PI-positive cells than HUVECs on SLA-Ti disks, which correlated with a pronounced reduction in their metabolic activity. Staining for macrophage subtype-specific markers showed the presence of CD163-positive macrophages on the LT-Ti disks, indicating the spontaneous switch towards a pro-regenerative phenotype. This finding that coupled with the hMSC and HUVEC results shows that the LT-Ti surface could enhance the bone–implant integration and improve long-term performance. In future studies, the behavior of further cell types involved in bone regeneration and surface topography, for example osteoclasts (cell type contributing to bone remodeling) as shown by Hefti et al. [63], need to be taken into account. Additionally, antibacterial response and regulation via different coatings and incorporation of nanoparticles within the LT-Ti surfaces should be addressed in follow-up research.

Based on the convincing results displayed by YH-hMSC osteogenesis upon culture on LT-Ti disks, we then assessed whether this surface could rescue hMSCs derived from aged or diseased bone (AH- and OP-hMSCs). During aging, the osteogenic capacity naturally decreases and even more with osteoporosis [64,65,66]. Primary hMSCs isolated from osteoporotic bone tissue have previously been demonstrated to have a number of pathological changes such as diminished cell growth, Collagen I expression and osteogenic potential [25,67,68]. Within this study, in vitro matrix mineralization by OP-hMSCs grown on polystyrene was hardly evident and ARS values around zero. Remarkably, both AH-hMSCs and OP-hMSCs reached significantly higher ARS levels when cultured on LT-Ti versus SLA and PS groups, demonstrating that sLST material holds great benefits for osteointegration in aged or osteoporotic bone. To further validate these exciting in vitro findings, in vivo experiments including not only healthy and aged but also osteoporotic animals will be part of the scope in future research. Osteoporotic pathology can be mimicked using well-established and characterized ovariectomy rat [69,70] and sheep models [71,72,73]. The novel LT-Ti implant materials can then be evaluated via metaphyseal osteotomy, resulting in the first insights on stable integration in low-density bone tissue.

With regards to testing and comparing the mechanical properties of LT-Ti and SLA-Ti, these were not subject of the current study. These properties depend on many factors including shape, cross-section, crystallinity, and grain size. In general, the presence of sharp-edged surface irregularities can lead to higher stress concentration in the surface layers of the material, which needs to be considered for SLA, as well as for LT treatment. Such surface changes could influence local plasticity, toughness, and ultimate tensile strength. The effect of SLA on fatigue properties of ultra-fine grained Ti grade 4 has been previously reported [74]. Also, the impact of laser texturing onto mechanical properties has been already studied [75], showing that laser texturing can increase the surface hardness and wear resistance of titanium surfaces by creating microstructural changes. Nevertheless, it may also introduce local residual stresses and can lead to localized degradation under load, which makes it obligatory to test mechanical properties upfront and conducting tests in vivo. The specific effects on mechanical properties like Young’s modulus and tensile strength vary significantly depending on the laser parameters (power, pulses, speed), texturing pattern, and titanium alloy used, and the above-mentioned factors. Therefore, examinations of mechanical characteristics will be conducted prior to testing the LT-Ti surfaces in in vivo experiments. Excitingly, with AI-based technologies rapidly developing, prediction of multiple biomaterial parameters in advance will likely not only become easier and more reliable [76] but also allow implant production to be adjusted according to the desired surface characteristics and anticipated function. Moreover, the AI advances in the area of computational models [77], such as in silico experiments, could also help to reduce the need of in vivo studies and might be applicable for forecasting the performance of LT-Ti in healthy versus osteoporotic bones.

## 5. Conclusions

The novel LT-Ti surface generated by sLST displayed a rough topography with distinctive micro- and nanodroplets, similar to natural bone tissue at the microscopic level. The overall biocompatibility, based on the response of bone-forming hMSCs, was as good as that of the current gold standard SLA-Ti. In terms of vascular and macrophage cell support, LT-Ti outperformed SLA-Ti with ca. 3-fold increase in metabolic activity for both cell types. Pilot RNA sequencing experiments, performed after 24 h of cell exposure to the material, revealed an interesting trend in surface-dependent responder genes. Most importantly, the LT-Ti surface significantly induced matrix mineralization not only by YH (ca. 6-fold higher), but also by AH and OP-hMSCs (ca. 10- to 24-fold). Taken together, these promising results may pave the way for developing next-generation titanium implants for uncemented fixation, with improved patient outcomes in both the elderly and osteoporotic populations.

## Figures and Tables

**Table 1:** General information on primary hMSCs cohorts.

	Young/Healthy (YH)	Aged/Healthy (AH)	Aged/Osteoporotic (OP)
Cohort size	*n* = 10	*n* = 5	*n* = 5
Age	27 ± 6 years	79 ± 5 years	76 ± 5 years
Gender	5 female, 5 male	5 female	5 female
Origin of cells	iliac crest	femoral head	femoral head
Surgery indication	iliac crest splint withdrawal	hip arthroplasty	hip arthroplasty
Inclusion criteria	age span	age span, osteoporotic status	age span, osteoporotic status
Exclusion criteria	Hepatitis, HIV, infection, neoplasia, drug or alcohol abuse, age < 18 years		

**Table 2:** Primer sequences for qRT-PCR.

Gene of Interest		Primer Forward (5′–3′) Primer Reverse (5′–3′)
*huCollagen1A1*	F R	acg tcc tgg tga agt tgg tc acc agg gaa gcc tct ctc tc
*huGAPDH*	F R	aat gaa ggg gtc att gat gg aag gtg aag gtc gga gtc aa

**Table 3:** Top 20 DEGs (FC > ±2 with *p* < 0.05) of SCP-1 cells on LT-Ti versus SLA-Ti.

Gene	Log_2_ Fold Change	*p*-Value	Adjusted *p*-Value	Additional Information
*TNFSF12-TNFSF13*	2.79	0.00880641	0.21904	tumor necrosis factor (ligand) superfamily, member 12/13; previously associated with osteoclastogenesis [30]
*H4C11*	2.66	0.01333683	0.24083	H4 clustered histone 11
*SULT1A4*	2.37	0.01240547	0.23682	sulfotransferase family, cytosolic, 1A, phenol-preferring, member 4; involved in catecholamine metabolism [31]
*TPTEP2-CSNK1E*	2.25	0.03866125	0.29592	readthrough, transmembrane phosphoinositide 3-phosphatase and tensin homolog 2 pseudogene and casein kinase I isoform epsilon
*DUSP6*	1.84	0.00000011	0.00178	dual specificity phosphatase 6; associated with osteoporosis [32]
*GJB2*	1.55	0.00492819	0.19375	gap junction beta-2
*RASD*	1.52	0.02925481	0.27577	Ras superfamily
*SHH*	1.47	0.0024782	0.18531	sonic hedgehog; hedgehog signaling important in bone formation [33]
*FGF12*	−1.48	0.01088293	0.22951	fibroblast growth factor 12
*WDR97*	−1.55	0.00835453	0.21905	WD Repeat Domain 97
*RHBDL1*	−1.55	0.01460635	0.24274	rhomboid like 1, enzyme coding
*TNFRSF6B*	−1.58	0.01454042	0.24274	TNF receptor superfamily member 6b
*SPAG17*	−1.68	0.0056264	0.20284	sperm-associated antigen 17; deficiency can lead to bone abnormalities [34]
*KCNIP2*	−1.7	0.04202795	0.30681	voltage-gated potassium channel-interacting protein 2
*ADGRF3*	−1.73	0.02897821	0.27577	adhesion G protein-coupled receptor F3
*NTS*	−1.76	0.00066852	0.14249	neurotensin
*AMY2B*	−1.79	0.02745122	0.27282	amylase alpha 2B
*OR2A20P*	−1.8	0.00240866	0.18540	pseudogene, olfactory receptor family 2 subfamily A member 20
*SEPT5-GP1BB*	−3.01	0.03476062	0.28526	readthrough septin 5 and glycoprotein Ib (platelet), beta polypeptide; affiliated with the lncRNA class
*ANKRD20A2P*	−3.25	0.01412988	0.24205	pseudogene, ankyrin repeat domain 20 family member A2

## Data Availability

The original contributions presented in this study are included in the article/Supplementary Material. Further inquiries can be directed to the corresponding authors. RNA sequencing data and raw data related to this manuscript will be uploaded to a public repository upon publication of the manuscript. A preprint copy will be available at: https://doi.org/10.5281/zenodo.17338456. Data will be accessible via the Zenodo repository (https://doi.org/10.5281/zenodo.17338495, accessed on 15 September 2025).

## Supplementary Materials

The supporting information can be downloaded at: https://www.mdpi.com/article/10.3390/jfb16110401/s1. Figure S1. Chondrogenically differentiated pellets stained with DMMB. Representative image for each hMSC donor (*n* = 10). Purple color confirmed the presence of glycosaminoglycans and thus successfully differentiation. Figure S2. Oil Red O staining of fat vacuoles of adipogenically differentiated hMSCs. Representative image for each hMSC donor (*n* = 10) of stimulated and control conditions. Figure S3. Representative scatter plots of CD73/90/105 staining, each shown vs. CD44 staining. The table next to each plot lists the respective percentage of negative/positive cells (events) per quadrant. Scatter plots and tables were generated with FCSalyzer software. Figure S4. ARS values for single YH-hMSC donors stimulated on polystyrene, SLA-Ti and LT-Ti (dots show single donors, some donor overlap). Figure S5. SEM images of osteogenically stimulated YH-hMSCs on (A) LT-Ti and (B) SLA-Ti disks. Blue arrows indicate cell bodies, yellow arrow—the spherical crystalline mineral deposits, green arrows—Ti substrate in the background. The regions a1-3 and b1-3, framed with yellow dashed lines, are magnified to their right. Figure S6. GO (gene ontology) analysis for molecular function using the described low-stringent analysis approach for (A) biological process and (B) molecular function. Figure S7. KEGG (Kyoto Encyclopedia of Genes and Genomes) analysis using the non-stringent analysis approach. Figure S8. Alizarin Red S quantification of osteogenically differentiated AH- and OP-hMSCs (*n* = 5, r = 3 stimulated, r = 2 control). Representative macroscopic images of one (A) OP hMSC donor and (B) AH hMSC donor on LT-Ti and SLA-Ti after ARS staining and washing steps. Alizarin quantification data as shown in Figure 10 is here separated into separate graphs for better visualization: (C) polystyrene (y-axis 0–0.6 mM), (D) SLA-Ti (y-axis 0–0.6 mM) and (E) LT-Ti (y-axis 0–7 mM) surface. Figure S9. Response of HUVEC cell line (*n* = 1) to LT-Ti and SLA-Ti disks. (A) Representative images of live (green) and dead (red) staining of HUVECs after 5 days culture on LT-Ti or SLA-Ti disks (r = 1 disk/group). (B) Metabolic/proliferative activity of HUVEC cells on LT-Ti and SLA-Ti disks (mean ± SD of r = 2 disks/group, *n* = 1). (C) Representative images of CD31 staining and DAPI counterstaining of HUVECs on LT-Ti and SLA-Ti disks (r = 1 disk/group). White dashed lines in LT-Ti images indicate the overlay of two z-level acquisitions—on the bottom and on the rim of the pore. Table S1. DEGs uncovered with stringent analysis |LogFC| ≥ 1 and adjusted *p*-value < 0.05.

## Abbreviations

The following abbreviations are used in this manuscript:

AH	(cells from) aged/healthy (cohort)
ARS	Alizarin Red S (staining solution)
BSE	backscattered electrons
DEG	differentially expressed gene
EDX	energy dispersive X-ray (analysis)
FBS	fetal bovine serum
h	hour(s)
hMSCs	human mesenchymal stromal/stem cells
LT-Ti	laser-textured titanium disk
min	minute(s)
OP	(cells from) aged/osteoporotic (cohort)
PS	polystyrene
RT	room temperature
SEM	scanning electron microscopy
SLA	sand-blasted and acid-etched
SLA-Ti	SLA-treated titanium disk
sLST	shifted laser surface texturing
YH	(cells from) young/healthy (cohort)
