# Supplementary material for: Micropatterning and Nanodropletting of Titanium by Shifted Surface Laser Texturing Significantly Enhances In Vitro Osteogenesis of Healthy and Osteoporotic Mesenchymal Stromal Cells
Source: J Funct Biomater. 2025 Oct 27;16(11):401. doi: 10.3390/jfb16110401 (PMC12653856; doi:10.3390/jfb16110401)
Supplement: Supplementary file 1 [file jfb-16-00401-s001.zip › jfb-3813167-supplementary.pdf]

Journal of Functional Biomaterials

MDPI

Supporting information

Micropatterning and Nanodropletting of Titanium by Shifted Surface Laser Texturing Significantly Enhances in vitro Osteogenesis of Healthy and Osteoporotic Mesenchymal Stromal Cells

Theresia Stich*, Francisca Alagboso, Girish Pattappa, Jin Chu, Denys Moskal, Michal Povolný, Maximilian Saller, Veronika Schönitzer, Konstantin J. Scholz, Fabian Cieplik, Volker Alt, Maximilian Rudert, Tomáš Kovářík, Tomáš Křenek and Denitsa Docheva*

Scheme 1. Materials and methods.

S1.1 Chondrogenic differentiation of YH-hMSCs

For chondrogenic differentiation, YH-hMSCs (n=10, r=4 pellets/donor) were seeded at 2x10^5 cells/well in V-bottomed 96-well plates. The plate was centrifuged at 500 x g for 5 min in order to allow the formation of cell pellets, which were then cultured for 28 days in chondrogenic differentiation medium (medium change thrice/week). The medium consisted of DMEM high-glucose (Gibco, Darmstadt, Germany), 10 % ITS+3 liquid media supplement (mixture of insulin, human transferrin (partially iron-saturated), sodium selenite, linoleic acid, and bovine serum albumin (BSA)), 10 nm dexamethasone, 50 µg/mL L-ascorbic acid, 10 ng/ml transforming growth factor β1 and 1 mM sodium pyruvate (all Sigma-Aldrich, Darmstadt, Deutschland). After 28 days, pellets were fixed, cryo-protected and embedded in TissueTek (Sakura Finetek, Umkirch, Germany) and cut into 10 µm sections using a cryotome (CM1950, Leica Biosystems GmbH, Nußloch, Germany). Sections on glass slides were washed in distilled water and afterwards placed in DMMB (dimethyl methylene blue) staining solution for 2 min at RT. Staining was microscopically evaluated with an Eclipse TE2000 microscope equipped with a Nikon camera (Nikon, Düsseldorf, Germany).

S1.2 Adipogenic differentiation of YH-hMSCs

Cells (1x10^4 cells/cm2) were seeded in 12-well-plates (n=10, r=3 wells/donor). Adipogenic stimulation medium consisted of DMEM high-glucose supplemented with 10 % FBS (PAN Biotech, Aidenbach, Germany), 1 % penicillin-streptomycin, 1 µM dexamethasone, 0.005 mg/ml insulin and 0.5 mM 3-isobutyl-1-methylxanthin (all Sigma-Aldrich). Cells were cultured for 21 days with media change twice/week. Afterwards, cells were first washed with 1xPBS (Gibco), fixed in 10 % formaldehyde in PBS (5 min, RT), rinsed with 60 % isopropanol and air dried for several minutes. Staining with 0.5 % Oil Red O solution in isopropanol took place for 10 min at RT. Four washing steps with Millipore H2O were applied prior to microscopic analysis as in S1.1.

S1.3 Culture of HUVEC cell line

HUVEC-TERT cell line (n=1; ATCC, Manassas, Virginia, USA) was propagated in HUVEC expansion media supplemented with 2 % FCS, 5 ng/mL vascular endothelial growth factor, 5 ng/mL endothelial growth factor and 5 ng/mL basic fibroblast growth

factor (all ATCC). After reaching confluence, passage 2 HUVECS were seeded onto LT-Ti and SLA-Ti disks (12 x 10^4 cells/disk) and cultured in HUVEC expansion medium for the remaining culture period. At each time point, two disks (r=2) per condition were used for resazurin assay described in 2.5.

S1.4 Immunocytochemistry

HUVECs on LT-Ti and SLA-Ti disks (12x10^4 cells/disk; r=1/group) were fixed in 4% formaldehyde PBS for 10 min at RT and washed thrice with 1xPBS. Disks were then incubated in blocking buffer (1 % bovine serum albumin, 10 % goat serum, 0.3 M glycine in 0.1 % PBS-Tween; all Sigma-Aldrich) for 1 h at RT, washed in PBS and incubated with CD31 antibody (1:50 dilution in blocking buffer, Cat. Nr. ab32457, Abcam, Cambridge, UK) overnight at 4 °C. Next, goat anti-rabbit FITC-conjugated secondary antibody (1:200 in PBS, Jackson, Maine, USA) was applied for 1 h at RT.

CD68 (M1) and CD163 (M2) staining was used for identifying macrophage types that were seeded on titanium disks. Macrophage seeded disks were fixed in 4% paraformaldehyde for ten minutes at room temperature and washed three times with PBS. Disks were then incubated in blocking buffer (1 % bovine serum albumin (Sigma-Aldrich), 10% goat serum (Sigma-Aldrich), 0.3 M glycine in 0.1 % PBS-Tween) for one hour at room temperature. Following a PBS wash, disks were incubated with either CD68 (1:50 dilution, Abcam, ab32457, Cambridge, UK) or CD163 (1:50 dilution, Abcam, ab87099, Cambridge, UK) antibodies in a blocking buffer overnight at 4 °C. The following secondary antibodies were used for one hour at room temperature: for CD163 goat anti-rabbit FITC antibody (1:200 in PBS, Jackson laboratories), whilst for CD68 goat anti-mouse FITC antibody (1:200 in PBS, Jackson laboratories).

For both cell types, the final step was incubation with 4,6-diamidino-2-phenylindole (DAPI, 1:100,000 in PBS) for 5 min and mounting with Mowiol anti-fading media. Afterwards, cells were imaged with a AxioObserver 7 fluorescence microscope equipped with a Axiocam 503 color camera (Zeiss, Oberkochen, Germany).

S2 Results

S2.1 Validation of chondrogenic lineage potential of YH-hMSCs

Chondrogenic differentiation of all ten donors was successful as judged by the positive DMMB staining, which causes a metachromatic shift from color blue to purple and thus, visualized glycosaminoglycans in the pellets. Donor variation was observed. (Figure S1).

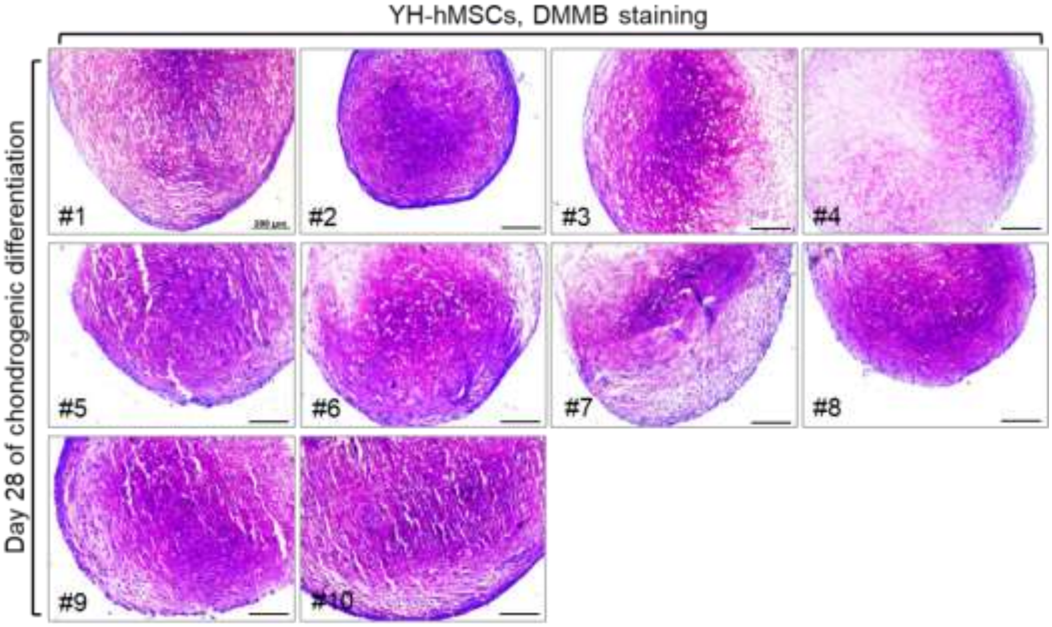

Figure S1. Chondrogenically differentiated pellets stained with DMMB. Representative image for each hMSC donor (n=10). Purple color confirmed the presence of glycosaminoglycans and thus successfully differentiation.

S2.2 Validation of adipogenic lineage potential of YH-hMSCs

Adipogenic differentiation was also validated for all ten donors via Oil Red O staining of lipid vacuoles (Supplementary Figure S2).

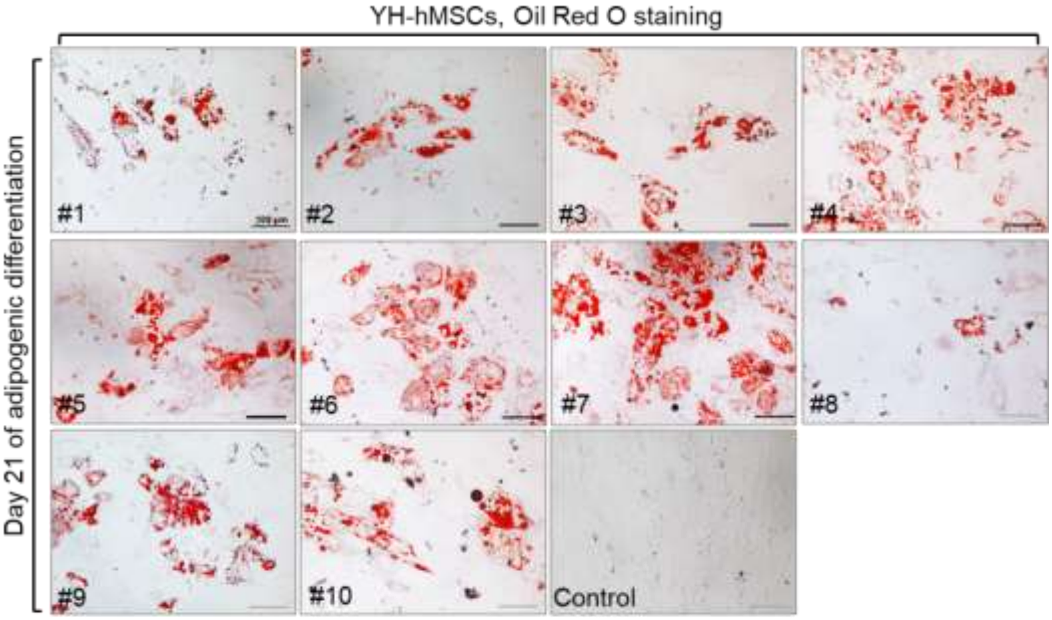

Figure S2. Oil Red O staining of fat vacuoles of adipogenically differentiated hMSCs. Representative image for each hMSC donor (n=10) of stimulated and control conditions.

S2.3 Scatter plots of flow cytometric analyses of YH-hMSCs

Cells were stained with mouse anti-human antibody fluorescence labels against the MSC markers CD73 (APC-A), CD90 (FITC) and CD105 (PerCP-Cy5-5-A). Simultaneously, as a common mesenchymal marker, cells were also stained with mouse anti-human CD44-PE-A-labelled antibody (all antibodies are included in the BD Stemflow hMSC Analysis

Kit, Cat.Nr. 562245, BD Biosciences, Heidelberg, Germany). Figure S3 shows, representative for one YH donor, scatter plots for the three positive markers, each versus CD44 staining. The tables next to each plot give an overview of the number and percentage of cells/events in each of the four quadrants. For all positive markers, > 98 % positive cells were achieved. Average values of n=6 analyzed cell samples are presented in Figure 4 in the main manuscript.

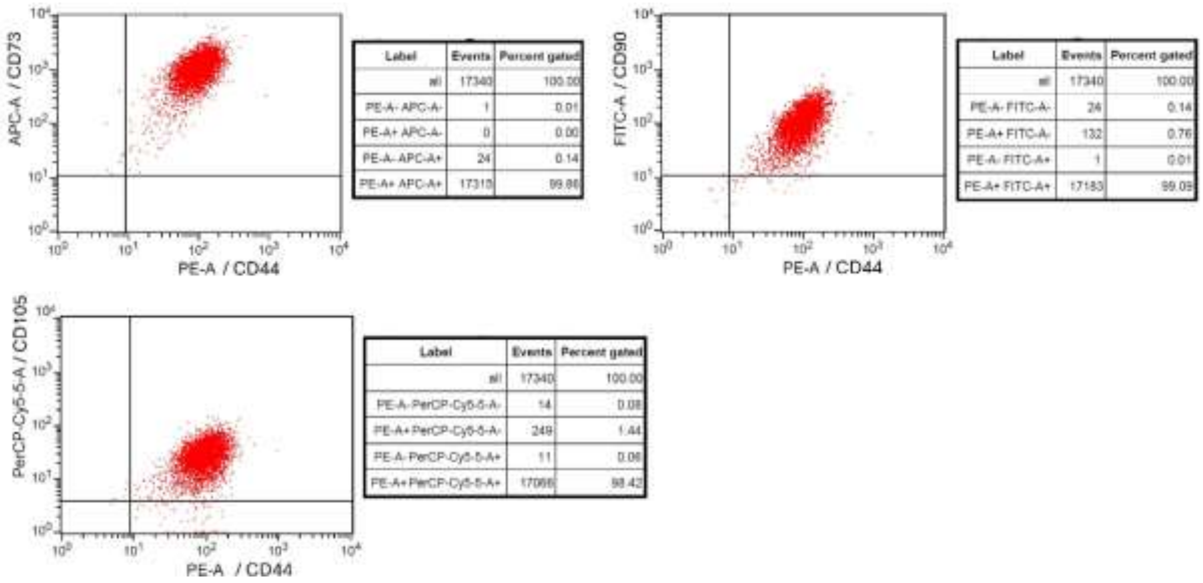

Label	Events	Percent gated
all	17340	100.00
PE-A- APC-A-	1	0.01
PE-A+ APC-A-	0	0.00
PE-A- APC-A+	24	0.14
PE-A+ APC-A+	17315	99.86

Label	Events	Percent gated
all	17340	100.00
PE-A- FITC-A-	24	0.14
PE-A+ FITC-A-	132	0.76
PE-A- FITC-A+	1	0.01
PE-A+ FITC-A+	17183	99.09

Label	Events	Percent gated
all	17340	100.00
PE-A- PerCP-Cy5-5-A-	14	0.08
PE-A+ PerCP-Cy5-5-A-	249	1.44
PE-A- PerCP-Cy5-5-A+	11	0.06
PE-A+ PerCP-Cy5-5-A+	17066	98.42

Figure S3. Representative scatter plots of CD73/90/105 staining, each shown vs. CD44 staining. The table next to each plot lists the respective percentage of negative/positive cells (events) per quadrant. Scatter plots and tables were generated with FCSalyzer software.

S2.4 Individual donor behavior for osteogenic differentiation on LT-Ti, SLA-Ti and PS

The graph in Figure S4 depicts individual donor differences in terms of mineralization, shown by quantification of ARS staining. All donors benefit in terms of osteogenic capacity when stimulated on LT-Ti.

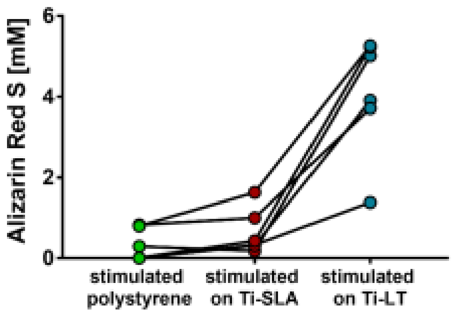

Figure S4. ARS values for single YH-hMSC donors stimulated on polystyrene, SLA-Ti and LT-Ti (dots show single donors, some donor overlap).

S2.5 SEM/BSE imaging of crystalline mineral deposits

To verify the presence of mineralized matrix of cells stimulated on LT-Ti and SLA-Ti, SEM/BSE technology was applied and results are shown in Figure S5.

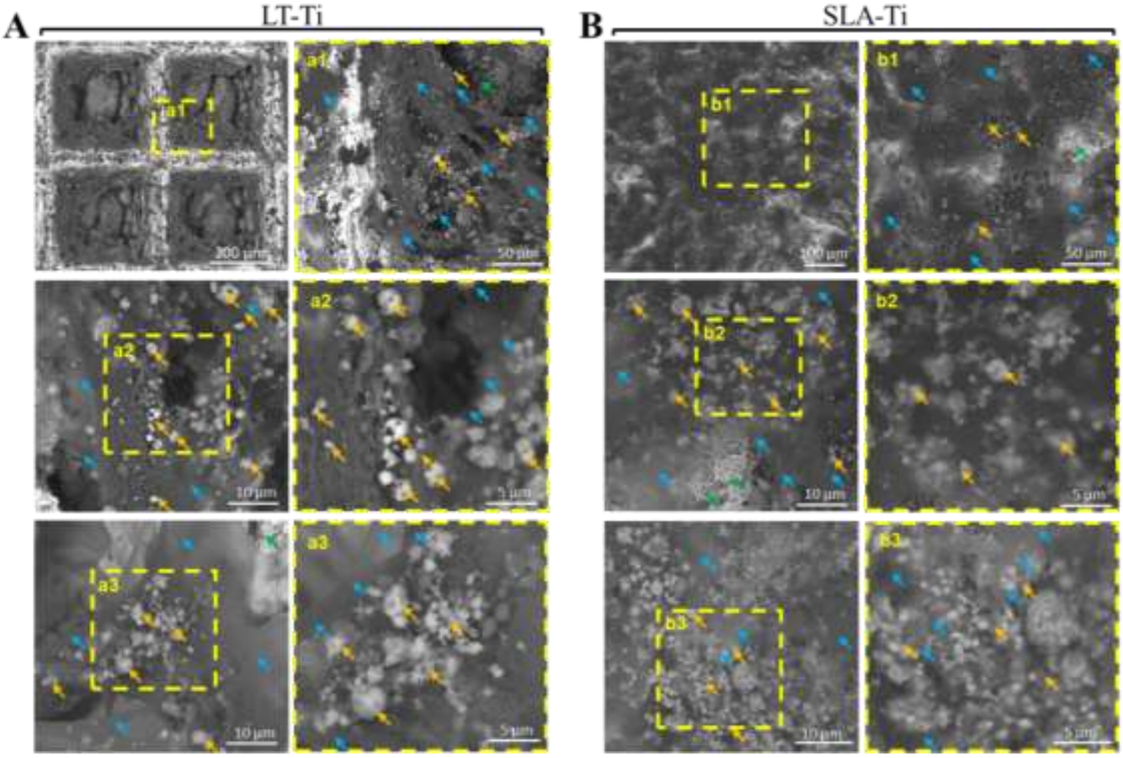

Figure S5. SEM images of osteogenically stimulated YH-hMSCs on (**A**) LT-Ti and (**B**) SLA-Ti disks. Blue arrows indicate cell bodies, yellow arrow - the spherical crystalline mineral deposits, green arrows - Ti substrate in the background. The regions a1-3 and b1-3, framed with yellow dashed lines, are magnified to their right.

S2.6 Bulk RNA-sequencing

Genes identified using the stringent analysis approach with a combination of LogFC and adjusted p-value are shown in Supplementary Table S1. Graphs for gene enrichment analyses GO and KEGG are in Figures S6 and S7.

Table S1. DEGs uncovered with stringent analysis |LogFC|≥1 and adjusted p-value < 0.05.

gene name	log2 fold change	p-value	adjusted p-value
**DUSP5**	1.058	1.527e-17	6.666e-14
**PHLDA1**	1.026	3.621e-20	2.371e-16
**DUSP6**	1.555	3.038e-34	3.978e-30
**ANGPTL4**	1.115	3.772e-17	1.235e-13
**SPRY4**	1.115	1.898e-16	4.143e-13

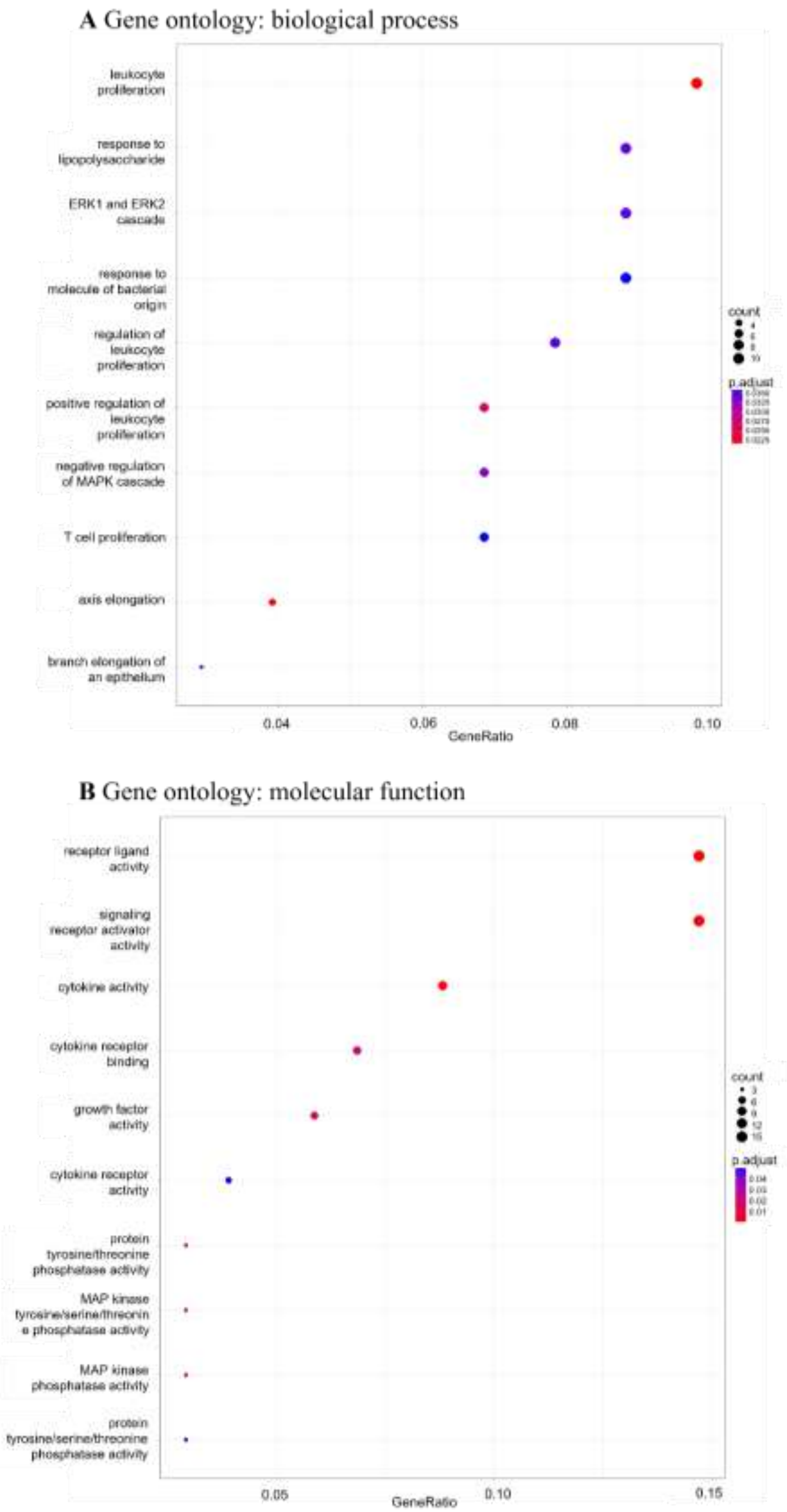

Figure S6. GO (gene ontology) analysis for molecular function using the described low-stringent analysis approach for (**A**) biological process and (**B**) molecular function.

GO analysis of the DEGs showed enrichment of gene clusters with the top accumulated terms "leukocyte proliferation", "response to lipopolysaccharide" and "ERK1 and

ERK2 cascade" for biological process "receptor ligand activity", "signalling receptor activator activity", "cytokine activity" and "cytokine receptor activity" for molecular function.

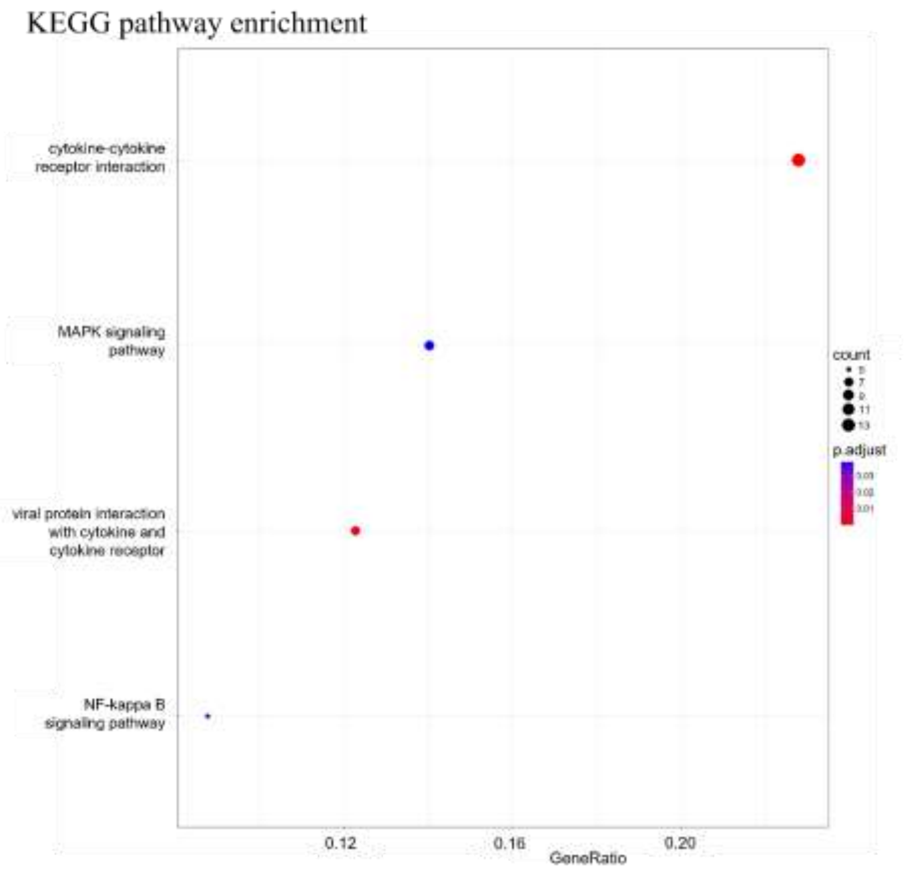

Figure S7. KEGG (Kyoto Encyclopedia of Genes and Genomes) analysis using the non-stringent analysis approach.

Subjecting all DEGs to KEGG signaling pathway analysis, "Cytokine-cytokine receptor interaction" was uncovered with the highest output, followed by "MAPK signalling pathway", "Viral protein interaction with cytokine and cytokine receptor" and "NF-kappa B signaling pathway."

S2.7 Alizarin Red S quantification of AH- and OP-hMSCs after 21 days of osteogenic stimulation/control cultivation

To enhance the visualization of the ARS quantification data for the three different cultivation surfaces (polystyrene, SLA-Ti and LT-Ti), the graphical data from Figure 10 is separated in into 3 graphs in Supplementary Figure S8 (C, D, E). Furthermore, representative images after ARS staining and washing steps of OP and AH hMSCs are shown in Supplementary Figure S8A, B.

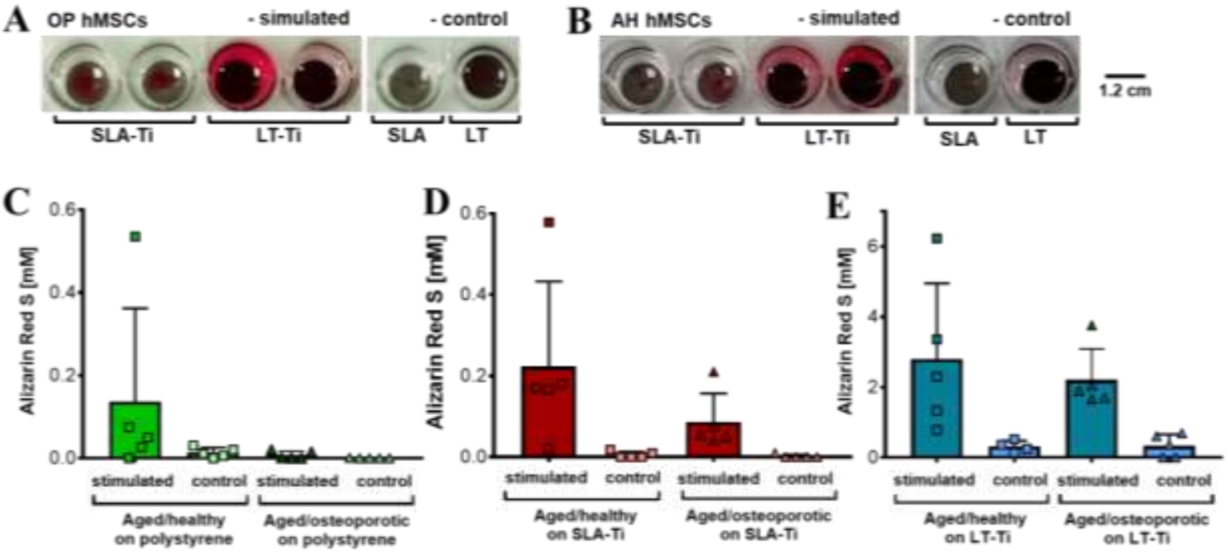

Figure S8. Alizarin Red S quantification of osteogenically differentiated AH- and OP-hMSCs (n=5, r=3 stimulated, r=2 control). Representative macroscopic images of one (**A**) OP hMSC donor and (**B**) AH hMSC donor on LT-Ti and SLA-Ti after ARS staining and washing steps. Alizarin quantification data as shown in Figure 10 is here separated into separate graphs for better visualization:(**C**) polystyrene (y-axis 0 – 0.6 mM), (**D**) SLA-Ti (y-axis 0 – 0.6 mM) and (**E**) LT-Ti (y-axis 0 – 7 mM) surface.

S2.7 Biocompatibility of LT-Ti and SLA-Ti for HUVEC cell line

Results described 3.10.

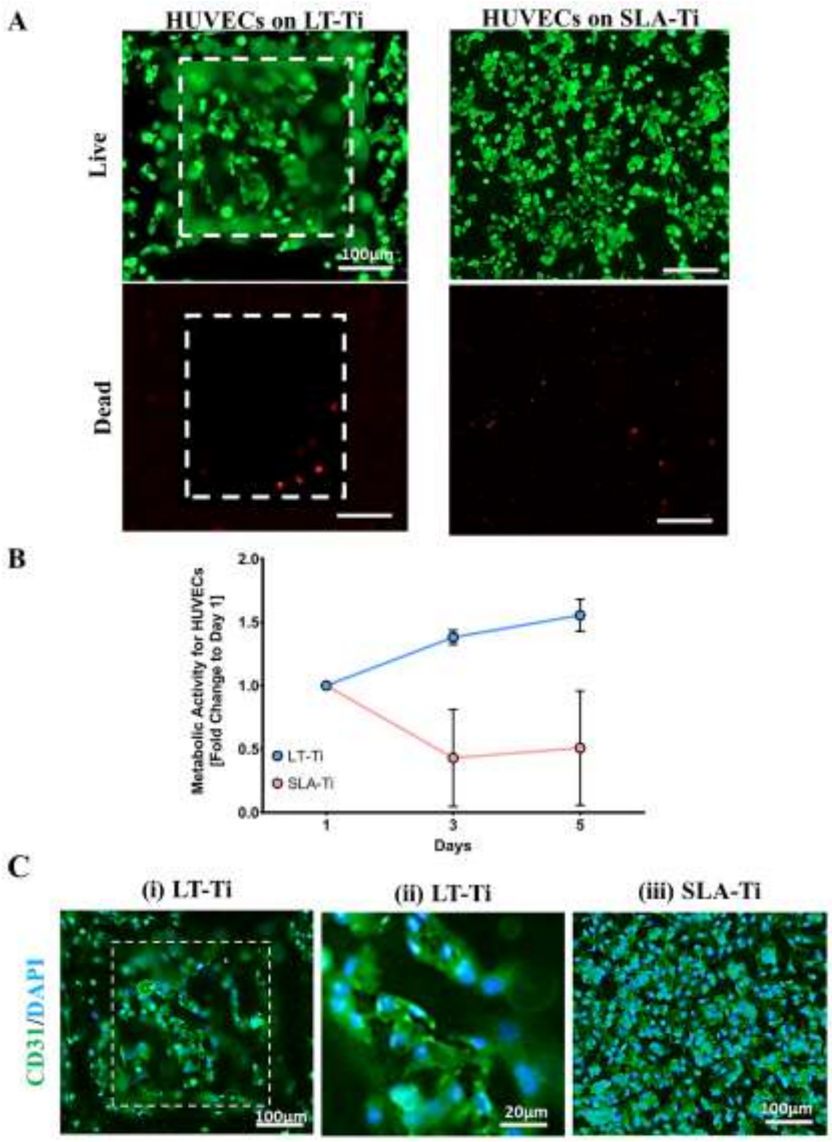

Figure S9. Response of HUVEC cell line (n=1) to LT-Ti and SLA-Ti disks. (**A**) Representative images of live (green) and dead (red) staining of HUVECs after 5 days culture on LT-Ti or SLA-Ti disks (r=1 disk /group). (**B**) Metabolic/proliferative activity of HUVEC cells on LT-Ti and SLA-Ti disks (mean +/- SD of r=2 disks/group, n=1). (**C**) Representative images of CD31 staining and DAPI counterstaining of HUVECs on LT-Ti and SLA-Ti disks (r=1 disk/group). White dashed lines in LT-Ti images indicate the overlay of two z-level acquisitions - on the bottom and on the rim of the pore.
